# NRP1 function and targeting in neurovascular development and eye disease

**DOI:** 10.1016/j.preteyeres.2016.02.003

**Published:** 2016-05

**Authors:** Claudio Raimondi, James T. Brash, Alessandro Fantin, Christiana Ruhrberg

**Affiliations:** UCL Institute of Ophthalmology, University College London, 11-43 Bath Street, London EC1V 9EL, UK

**Keywords:** Angiogenesis, Neovascularisation, Vascular permeability, Endothelial cell, Neuropilin, NRP1/2, neuropilin 1/2, VEGF, vascular endothelial growth factor, VEGFR1/2, vascular endothelial growth factor receptor 1/2, ERK1/2, extracellular signal-regulated kinases 1/2, AMD, age-related macular degeneration, PDR, proliferative diabetic retinopathy, ROP, retinopathy of prematurity, RVO, retinal vein occlusions, BRVO, branch RVO, CRVO, central RVO, DME, diabetic macular oedema, OIR, oxygen-induced retinopathy, CNV, choroidal neovascularisation, RPE, retinal pigment epithelium

## Abstract

Neuropilin 1 (NRP1) is expressed by neurons, blood vessels, immune cells and many other cell types in the mammalian body and binds a range of structurally and functionally diverse extracellular ligands to modulate organ development and function. In recent years, several types of mouse knockout models have been developed that have provided useful tools for experimental investigation of NRP1 function, and a multitude of therapeutics targeting NRP1 have been designed, mostly with the view to explore them for cancer treatment. This review provides a general overview of current knowledge of the signalling pathways that are modulated by NRP1, with particular focus on neuronal and vascular roles in the brain and retina. This review will also discuss the potential of NRP1 inhibitors for the treatment for neovascular eye diseases.

Neuropilin 1 (NRP1) is expressed in many cell types, including neurons and blood vessels, and constitutive NRP1 knockout mice are embryonically lethal with both neural and vascular defects (Kawasaki et al., 1999, Kitsukawa et al., 1997, Lampropoulou and Ruhrberg, 2014, Schwarz and Ruhrberg, 2010). Since its discovery in 1987 as a cell adhesion molecule termed A5 in the frog nervous system (Takagi et al., 1987), an excess of 1775 PubMed citations have become available that have either examined the structure, expression or function of NRP1 in organ development or pathology. These studies have defined NRP1 roles in a range of signalling pathways that utilise diverse extracellular ligands. In particular, NRP1's ability to modulate vascular responses in tumour growth has sparked much interest in manipulating its function. Some of the emerging therapeutics to modulate NRP1 function have provided useful tools for experimental investigation and have clinical potential to treat ocular neovascular diseases such as retinopathy of prematurity (ROP), proliferative diabetic retinopathy (PDR) and age-related macular degeneration (AMD) (Foster and Resnikoff, 2005). This review provides a general overview of current knowledge of signalling pathways that are modulated by NRP1 and will discuss clinical potential of NRP1 targeting to treat neovascular eye disease.

## 1 Structure of the neuropilins (NRPs)

**Fig. 1:**
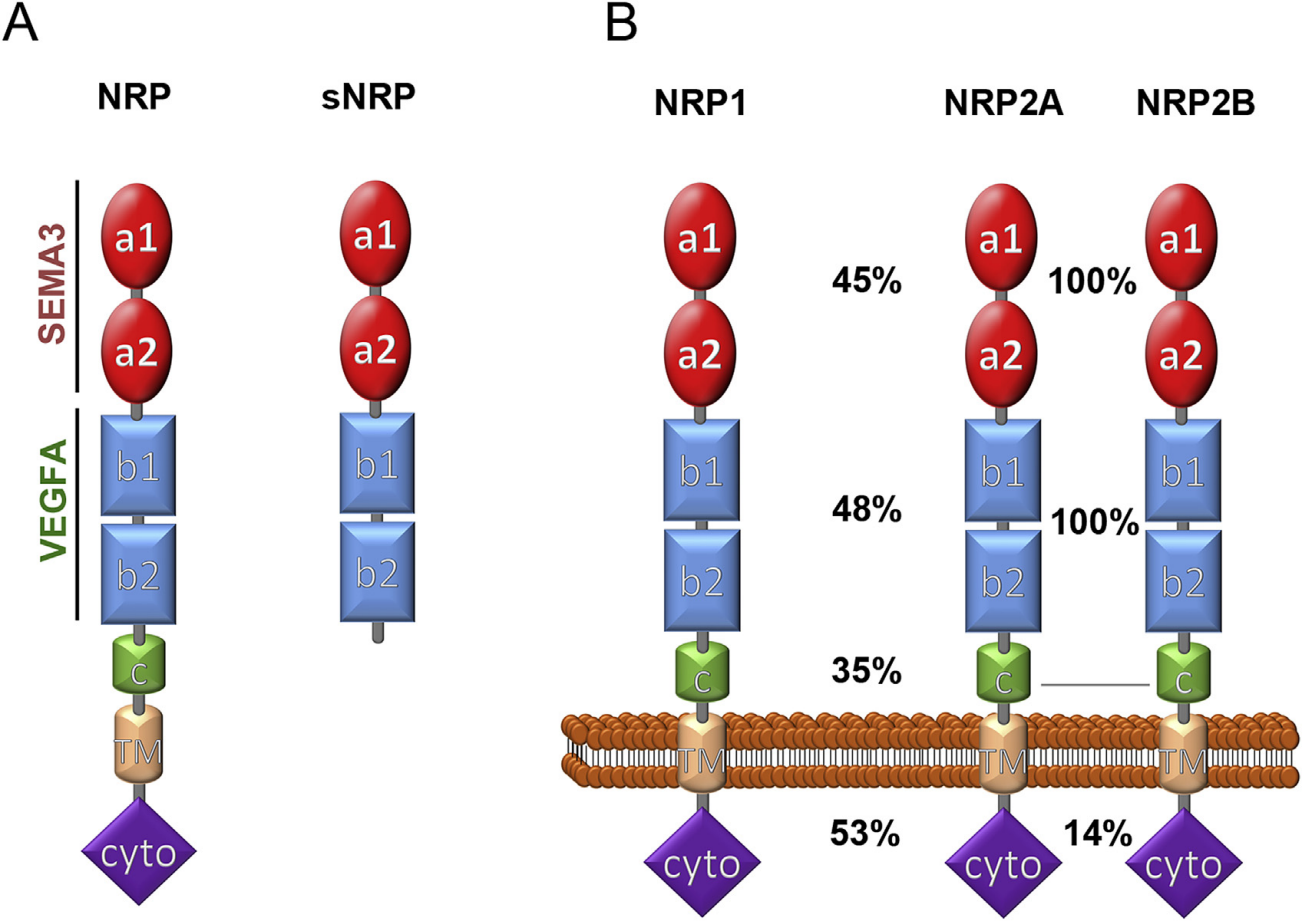
Structure of NRP1, NRP2 and their splice variants. (A) Schematic representation of the transmembrane and soluble forms of both NRPs. Transmembrane NRP forms consist of seven domains, two complement (CUB) domains (a1 and a2), two coagulation factor (FV/FVIII) domains (b1 and b2), a MAM domain (c), a transmembrane region (TM) and a cytoplasmic (cyto) domain that interacts with intracellular proteins containing a PDZ domain. Soluble NRPs have the a and b domains, but lack the transmembrane and cytoplasmic domains. (B) Amino acid identity between corresponding domains in transmembrane NRP1 and the NRP2 splice variants NRP2a and NRP2b; the grey line indicates the position after which NRP2A and NRP2B have no more homology.

Both NRP1 and its homolog NRP2 are glycoproteins encoded by genes that are alternatively spliced into full-length transmembrane receptors and shorter soluble forms (Gagnon et al., 2000) (Fig. 1). The NRP1 transmembrane form is encoded by 17 exons and composed of an extracellular domain of about 840 amino acids, a single-pass transmembrane domain of 23 amino acids and a 44 amino acid cytoplasmic domain (Schwarz and Ruhrberg, 2010). The importance of NRP1 for a host of diverse developmental and pathological processes can be explained by its organisation into several structurally distinct domains (Fig. 1A) that mediate interactions with many different other proteins and are alternatively spliced. The extracellular NRP1 part consists of two domains called a1 and a2, which resemble the CUB (complement, Uegf, BMP) domain present in complement components. They are followed by the b1 and b2 domains, which are similar to coagulation factor V/VIII domains. The c domain, with homology to a MAM (meprin/antigen 5/receptor tyrosine phosphatase μ domain), separates the other extracellular domains from the transmembrane region. The short intracellular (cytoplasmic) domain is catalytically inactive, but contains a C-terminal SEA (serine-glutamine-alanine) motif that interacts with intracellular proteins containing a PDZ domain (Schwarz and Ruhrberg, 2010).

NRP2 was identified based on its homology to NRP1 (Chen et al., 1997). The amino acid sequences of the corresponding a, b and c domains of human NRP1 and NRP2 are 45%, 48%, and 35% identical (Fig. 1B). Two NRP2 alternative splicing variants exist, NRP2A and NRP2B (Gu et al., 2003, Nakamura et al., 2000, Schwarz and Ruhrberg, 2010) (Fig. 1B). Human NRP2A and NRP2B have identical a, b, and c domains, but their sequence differs after amino acid 808, which localises in the linker region connecting the c domain and transmembrane domains. The NRP2A cytoplasmic domain shares 53% identity with the NRP1 cytoplasmic domain and also has a SEA motif, whereas the NRP2B cytoplasmic domain lacks the SEA motif and is unable to interact with PDZ domain containing proteins (Rossignol et al., 2000).

Soluble forms of both NRP1 and NRP2 have also been described (Fig. 1A). The soluble forms s_11_NRP1 and s_12_NRP1 are encoded by the first 11 and 12 exons of the *NRP1* gene, respectively, and s_9_NRP2 is encoded by the first 9 exons of the *NRP2* gene (Gagnon et al., 2000, Rossignol et al., 2000). More recently, transcripts for two additional soluble isoforms, named s_III_NRP1 and s_IV_NRP1, were identified in a human expression sequence tag (EST) clone library (Cackowski et al., 2004). s_III_NRP1 contains the sequence encoded by the first 9 exons and exon 12, but skips exons 10 and 11, whereas the s_IV_NRP1 mRNA contains the first 10 exons and exon 12, but lacks exon 11. As all soluble NRP1 isoforms lack the c, transmembrane and cytoplasmic domains (Fig. 1A), they can bind NRP1 ligands, but are unable to transduce signals and thus may serve as decoy receptors to sequester NRP1 ligands (Gagnon et al., 2000). Whilst soluble NRP1 is expressed in cells of the liver and kidney (Gagnon et al., 2000), little is known about its endogenous functions. In contrast, transmembrane NRP1, but not soluble NRP1 is expressed in blood vessels (Gagnon et al., 2000). Transmembrane NRP1 has been implicated in the development and function of numerous tissues, most notably blood vessels and neurons. The multiple neurovascular functions of the transmembrane isoform of NRP1 are therefore the main topic of this review.

### 1.1 The NRP1 extracellular domain has a modular structure to bind diverse ligands

Even though NRP1 was originally identified as an adhesion molecule in the nervous system (Takagi et al., 1995), it has since been studied primarily as a receptor for the class 3 semaphorin (SEMA3) A, a secreted glycoprotein that regulates axon guidance (He and Tessier-Lavigne, 1997, Kolodkin and Ginty, 1997), and as a receptor for specific isoforms of the vascular endothelial growth factor A (VEGF) (e.g. Soker et al., 1996, Soker et al., 1998). Similarly, NRP2 has been described both as a SEMA3 family and VEGF isoform receptor (e.g. Chen et al., 1997, Giger et al., 1998, Gluzman-Poltorak et al., 2000).

The SEMA3 class of secreted semaphorins is comprised of SEMA3A, SEMA3B, SEMA3C, SEMA3D, SEMA3E, SEMA3F and SEMA3G (reviewed in Neufeld et al., 2012). Whereas NRP1 predominantly binds SEMA3A as well as SEMA3C, NRP2 preferentially binds SEMA3F and SEMA3C, but also SEMA3B, SEMA3D and SEMA3G (reviewed in Raper, 2000, Sharma et al., 2012). Even though NRP1 acts mainly as a SEMA3A receptor and NRP2 as a SEMA3F receptor, there are exceptions to this general rule (see Section 5).

The *VEGFA* gene consists of 8 exons and encodes three major alternatively spliced isoforms termed VEGF189, VEGF165 and VEGF121 in humans and VEGF188, VEGF164 and VEGF120 in mice, respectively, with the numbers indicating the number of amino acids in the mature polypeptide (reviewed in Ruhrberg, 2003). These isoforms differ by the presence or absence of protein domains expressed by exon 6 and 7, with VEGF189 containing both domains and VEGF165 containing the domain encoded by exon 7 and VEGF121 lacking both the exon 6 and 7 domains. Exons 6 and 7 as well as the shared C-terminal exon 8 have all been implicated in VEGF isoform binding to NRPs. As described in the following paragraphs, structure-function studies of NRP1 have shown that binding to these diverse ligands is mediated by the a and b domains.

The a1 and a2 domains confer SEMA3 binding specificity, and NRP1 mutant proteins lacking the a1 or a2 domains fail to bind SEMA3A *in vitro* (Gu et al., 2002). In contrast, these SEMA3A binding-deficient mutants retain the ability to bind VEGF165 (Gu et al., 2002). Point mutations have been introduced into the a1 domain to create a mouse mutant that lacks SEMA3A-, but not VEGF165-binding to NRP1 (Gu et al., 2003). Analysis of these mice has provided much insight into NRP1 function in the vascular and nervous systems (see Sections 3, 4, 5). The b1 domain additionally contributes to the binding of SEMA3 proteins that have been proteolytically cleaved by furin endopeptidases to expose a C-terminal arginine that binds the b1-b2 domain (Gu et al., 2002, Parker et al., 2010, Parker et al., 2012a).

VEGF165 binds to NRP1 via the b1 and b2 domains (Gu et al., 2002, Mamluk et al., 2002). Whereas loss of the b1 domain abrogates VEGF165 binding to NRP1, loss of the b2 domain only reduces NRP1 affinity for VEGF165 (Gu et al., 2002). Recent evidence showed that NRP1 b1 domain mutants with point mutations replacing tyrosine residue 297 with alanine (Y297A) or aspartate residue 320 with lysine or alanine (D320K or D320A) are unable to bind VEGF165, but maintain SEMA3A binding (Gelfand et al., 2014, Herzog et al., 2011).

Although VEGF165 is the main VEGF-A isoform that binds NRP1 (Soker et al., 1998), VEGF121 and VEGF189 are also capable of binding NRP1. VEGF121 binding to the NRP1 b1 domain is mediated by the exon 8-encoded domain, which carries a C-terminal arginine residue (Pan et al., 2007b, Parker et al., 2012b). However, the affinity of VEGF121 for NRP1 is 10 times lower than that of VEGF165 (Parker et al., 2012b). In agreement, in situ ligand-binding assays with alkaline phosphatase-conjugated VEGF isoforms on intact mouse brain tissue demonstrated that VEGF165 binds NRP1-expressing axon tracts *in vivo*, but that VEGF121 is unable to do so (Tillo et al., 2015, Vieira et al., 2007). Similar studies also showed that VEGF189 binds NRP1 (Tillo et al., 2015). Biochemical studies demonstrated that NRP1 VEGF189 has higher affinity than VEGF165 for NRP1, mainly due to a lower dissociation constant that may be explained by VEGF189 containing both the exon 6- and 7, as well as the exon 8-encoded domains (Vintonenko et al., 2011).

Recently, VEGF165b was identified as another alternative splice form of VEGF. VEGF165b is identical to VEGF165, except for the last six amino acids, which are encoded by an alternative exon 8, and it therefore has a different C-terminal domain (Bates et al., 2002). Although VEGF165b contains the exon 7-encoded domain, it does not interact with NRPs, demonstrating that the C-terminus encoded by exon 8 of the other VEGF isoforms is critical for its the interaction with NRPs (Vander Kooi et al., 2007).

Similar to NRP1, NRP2 binds VEGF165, and additionally, a less common VEGF isoform termed VEGF144 (Gluzman-Poltorak et al., 2000). However, NRP2 has a 50 fold lower affinity for VEGF165 compared to NRP1, likely due to the different amino acid sequence in the b1 domain (Parker et al., 2012b).

The NRP1 b1 and b2 domains additionally serve as binding sites for several other ligands, in particular those that share homology with VEGF, including VEGFB, VEGFC, VEGFD and the placental growth factor 2 PLGF2 (also known as PGF) (Karpanen et al., 2006, Makinen et al., 1999, Migdal et al., 1998). NRP1 also binds hepatocyte growth factor (HGF), several members of the fibroblast growth factor (FGF) family as well as latent and active transforming growth factor beta 1 (TGFβ1) (Glinka and Prud'homme, 2008, Hu et al., 2007, West et al., 2005). The ability of the NRP1 b1 domain to bind these proteins may be linked to its ability to function as a heparin mimetic that supports ionic bonding with the heparin-binding site of growth factors (West et al., 2005).

The b1 and b2 domains are also required for NRP1-mediated cell adhesion activity (Shimizu et al., 2000). The cell adhesion ligand for NRP1 is an unidentified protein distinct from SEMA3A or VEGF (Shimizu et al., 2000).

### 1.2 The cytoplasmic NRP1 domain recruits intracellular proteins

The 44 amino acid NRP1 cytoplasmic domain has no known catalytic activity, but contains a SEA motif that is able to recruit the PDZ domain-containing adaptor protein synectin (Gao et al., 2000), also known as GIPC1 (Cai and Reed, 1999). Synectin binds myosin VI to enable NRP1 trafficking into early endosomes (Lanahan et al., 2010). The NRP1 cytoplasmic domain has also been reported to interact with the non-receptor tyrosine kinase ABL1 in tumour cells (Yaqoob et al., 2012). The role of these NRP1 cytoplasmic domain interactions is discussed in detail below (Section 4.4.1 and Section 5.1). According to the phosphosite bioinformatics resource (www.phosphosite.org), an excess of 485 high throughput proteomic discovery-mode mass spectrometry analyses identified post-translational phosphorylation of human NRP1 at Y920, which is located next to the SEA domain. Post-translational modification of the NRP1 cytoplasmic tail may enable modulation of NRP1 signalling, but their existence has yet to be validated through conventional biochemical analyses.

### 1.3 NRPs form complexes with several receptors and intracellular adaptors

The c domain of NRP1 mediates homodimerisation and heterodimerisation with NRP2 (Gluzman-Poltorak et al., 2001, Herzog et al., 2011). Experiments in which the NRP1 transmembrane and cytoplasmic domains are absent or replaced by the corresponding NRP2 sequences suggest that the transmembrane domain and intracellular domain of NRP1 are not sufficient for dimerisation (Nakamura et al., 1998). In fact, it is the c and transmembrane domains that mediate dimer formation (Giger et al., 1998, Roth et al., 2008).

NRP1 and NRP2 interact with transmembrane proteins of the plexin (PLXN) family to convey semaphorin signals, with NRPs serving as the ligand binding and plexins serving as signal transducing subunits (reviewed in Schwarz and Ruhrberg, 2010). The cytoplasmic domain of plexins contains a GTPase-activating protein (GAP) domain that stimulates signal transduction, but a semaphorin-binding domain in the extracellular PLXN domain maintains PLXN in an inactive conformation unless the SEMA3/NRP complex is bound to the PLXN (Takahashi and Strittmatter, 2001).

NRP1 can also form VEGF165-dependent complexes with the VEGF receptor tyrosine kinase 2 (VEGFR2, also known as KDR or FLK1) (e.g. Soker et al., 2002). VEGF165 contains a cysteine knot motif encoded by exon 4 that contacts VEGFR2 in addition to its exon 7/8-encoded region that interacts with NRP1 to enable bridge formation between both receptors; hence, mutations of either Y297 or D320 in NRP1 exon 6, which impair VEGF165 binding, inhibit complex formation between NRP1 and VEGFR2 (Herzog et al., 2011). Even though VEGF121 has the exon 4-encoded domain and binds NRP1 via its exon 8-encoded domain, this interaction is not sufficient to enable complex formation between NRP1 and VEGFR2, at least *in vitro* (Pan et al., 2007b).

Similar to NRP1, NRP2 has been shown to interact with VEGFR2 in a VEGF165-dependent manner *in vitro* (Favier et al., 2006). Both NRP1 and NRP2 can also form a complex with VEGFR1 in cells overexpressing these proteins, and complex formation is stimulated by both VEGF165 and VEGF121 (Fuh et al., 2000, Gluzman-Poltorak et al., 2001). Additionally, NRP2 interacts with VEGFR2 and VEGFR3 in a VEGFC-dependent manner (Favier et al., 2006).

NRP1 has been observed to interact with integrins, receptors for extracellular matrix components such as fibronectin, laminin and collagen. Two integrins have been identified in endothelial cells, the fibronectin receptor α5β1 integrin and the vitronectin receptor αvβ3 (e.g (Dejana et al., 1990). *In vitro* studies showed that NRP1 interacts with the β1 subunit in human cancer and arterial endothelial cells (Fukasawa et al., 2007, Serini et al., 2003). Most recently, co-immunoprecipitation analysis of brain lysates revealed that NRP1 forms a complex with β8 integrin on neuroepithelial cells (Hirota et al., 2015). The functional significance of NRP1 interactions will be discussed in Section 4.1.

Similar to NRP1, NRP2 interacts with integrins in different cell types. Thus, immunoprecipitation analysis revealed that NRP2 interacts with α5 integrin in endothelial cells (Cao et al., 2013) and with α6β1 integrin in melanoma and breast cancer cells (Goel et al., 2012).

Both NRP1 and NRP2 form a complex with the TGFβ receptor 1 (TGFBR1) and the TGFβ receptor 2 (TGFBR2), independently of TGFβ1 binding (Glinka et al., 2011). Biochemical studies showed that NRP1 and NRP2 have a similar affinity for TGFBR1, whereas NRP2 has a higher affinity for TGFBR2 compared to NRP1 (Glinka et al., 2011). NRPs role in TGFβ signalling will be further discussed in Section 4.2.

In human mesenchymal stem cells, NRP1 also interacts with PDGFRα in the presence of PDGFA, PDGFB or VEGF165, and with PDFGRβ in the presence of PDGFB or VEGF165 (Ball et al., 2010). It therefore appears likely that these ligands enable bridge formation, akin to the role of VEGF165 in NRP1-VEGFR2 complex formation. NRP1 also forms a complex with PDGFRα in primary human aortic smooth muscle cells (Evans et al., 2011, Pellet-Many et al., 2011). NRPs role in PDGFR signalling will be further discussed in Section 4.3.

## 2 Models to study the functional significance of SEMA3 and VEGF signalling through NRP1

The functional significance of SEMA3 and VEGF signalling through NRP1 has been studied in several different contexts, including in neuronal and vascular development, tumourigenesis, ocular neovascular disease and immune system function (Chaudhary et al., 2014, Ellis, 2006, Fantin et al., 2012, Graziani and Lacal, 2015, Plein et al., 2014). This review will largely focus on current literature describing NRP1's neurovascular roles in development and disease, with specific reference to the brain and retina.

### 2.1 The mouse hindbrain and retina as models to study neurovascular development

The mouse embryo hindbrain and retina are widely used models for the qualitative and quantitative description of molecular and cellular mechanisms regulating neovascularisation. The hindbrain is vascularised early in development to support the growth of rapidly proliferating neural progenitors and the function of their progeny, and vascularisation follows a stereotypical process (reviewed by Ruhrberg and Bautch, 2013). Thus, vessels sprout from the perineural vascular plexus into the mouse hindbrain at around embryonic day (E) 9.75 towards the ventricular zone, and the first intraneural vascular network beneath the hindbrain ventricular zone is established by E12.5 (Fantin et al., 2010, Ruhrberg et al., 2002). Hindbrain vascularisation is described in more details in Section 5.

The retinal vasculature is comprised of three layers, a superficial, deep and intermediate plexus (reviewed by Fruttiger, 2002, Ruhrberg and Bautch, 2013). The superficial vascular plexus forms when vessels originating in the optic nerve invade the retina and grow centrifugally towards the retinal periphery during the first week after birth in rodents. From postnatal (P) day 7 onwards, the superficial vascular plexus sprouts vertically to form first the deep and then the intermediate vascular plexus (Fruttiger, 2002, Ruhrberg and Bautch, 2013). By P12, the deep plexus has reached the retinal periphery. This is followed by growth of the intermediate plexus in the inner plexiform layer, and by p21 the retinal vasculature is mature and efficiently perfuses the retina (reviewed in Fruttiger, 2002, Ruhrberg and Bautch, 2013, Stahl et al., 2010).

The hindbrain model is particularly useful to analyse angiogenesis in mouse mutants suffering midgestation lethality. In contrast, retinal angiogenesis in rodents occurs postnatally and is therefore not suitable to study embryonically lethal mouse mutants. However, both models can be adapted to study angiogenesis in inducible endothelial-specific conditional knockout mice to overcome embryonic lethality (Fantin et al., 2013b, Plein et al., 2015b). Both the hindbrain and retina models have been used extensively to define the function of NRP1 and its ligands in developmental angiogenesis (see Section 5).

### 2.2 Neovascular eye diseases and relevant mouse models

Ocular neovascularisation causes visual loss in several diseases including ROP, PDR, BRVO and exudative AMD (reviewed in Campochiaro, 2013, Campochiaro, 2015). The role of disregulated VEGF or SEMA signalling through NRP1 in these conditions will be discussed in subsequent chapters following a brief overview of these conditions and relevant mouse models.

ROP occurs in premature neonates that receive oxygen therapy during intensive care because of their immature lungs. Increased oxygen levels destabilise the immature developing vasculature of the retina, and the ensuing vascular regression causes hypoxia on return to room air. Hypoxia then stimulates the growth of neovessels that are fragile, leaky and protrude into the vitreous, causing haemorrhage, retinal scarring and retinal detachment (Campochiaro, 2015). In PDR, a leading cause of blindness in adults of working age, diabetic metabolic syndrome causes retinal capillary degeneration and occlusions which result in retina ischemia. The ischemic tissues increase the synthesis of pro-angiogenic factors, which stimulate the growth of abnormal leaky vessels from pre-existing retinal venules, promoting the development of diabetic macular oedema (DME). The mouse oxygen-induced retinopathy (OIR) model has many hallmarks of human ROP and PDR (Smith et al., 1994). In the OIR model, the exposure of neonatal mice to hyperoxia induces vaso-obliteration of central retinal capillaries, which on subsequent return of mice to normoxia causes tissue hypoxia and neovascularisation. Whilst there is some revascularisation of avascular areas, neovascular pathology is characterised by tuft-like vascular malformations from veins and capillaries that have escaped regression (Connor et al., 2009, Smith et al., 1994). These vascular malformations protrude into the vitreous instead of re-establishing a functional vascular network in the ischemic areas of the retina. The OIR model has been used recently to define the function of NRP1 and its ligands in retinal neovascularisation, whilst a mouse model of diabetes has also been used to study the role of NRP1 and its ligands in diabetic retinopathy and ocular oedema (Section 6).

Retinal vein occlusions (RVO) are the second most common type of retinal vascular disorder after diabetic retinal disease, can occur at almost any age, but mostly in patients aged over 50, and they cause sudden unilateral loss of vision (Rogers et al., 2010). While central RVO (CRVO) results from thrombosis in the central retinal vein within the optic nerve, branch RVO (BRVO) occurs at more distal sites due to focal occlusion of a retinal vein at an arterio-venous crossing-point, where compression of the vein by an artery passing anteriorly produces turbulence and thrombosis (Cahill and Fekrat, 2002). Loss of vision in these conditions is usually secondary to neovascularisation and retinal oedema (Campochiaro, 2015). A mouse model of BRVO has recently been developed, which involves laser-induced photocoagulation of a retinal vein after systemic injection of rose bengal (Zhang et al., 2007). Several different genetically engineered mouse mutants have been reported to increase the frequency of arteriovenous crossings in the retina, including some affecting NRP1 (see section 4.4.1). As arteriovenous crossings in the retina are a known risk factor for BRVO (Weinberg et al., 1990, Zhao et al., 1993), these mouse models may be useful to study the pathogenesis of BRVO.

AMD is the leading cause of severe visual loss in people over the age of 65. In contrast to ROP and PDR, in which retinal neovasculature invades the vitreous cavity, neovascularisation in AMD originates from the choroidal vasculature and extends into the subretinal space of the macula, the area of retina responsible for central vision, thus causing loss of vision (reviewed in Campochiaro, 2015). A model of choroidal neovascularisation (CNV) induced by laser injury of the Bruch's membrane that separates the choroidal vasculature from the retinal pigment epithelium (RPE) and the neural retina exist in the mouse. In this model, the laser injury ruptures the outer blood-retina barrier, causes inflammation and induces CNV towards the neural retina and vascular leakage (Balaggan et al., 2006). Even though a recent report linked genetic variation in *NRP1* to treatment response in patients with neovascular AMD (see Section 6), NRP1 function has not yet been studied in the CNV model.

## 3 Functional significance of SEMA3 signalling though NRPs

SEMA3 signalling through NRPs has been shown to regulate a multitude of cell behaviours, in particular in the central and peripheral nervous system, but also in the cardiovascular and immune systems. Cell type- and context-specific cellular responses are achieved, in part, through the association of the NRPs with different plexins and the contribution of specific intracellular signal transduction pathways (reviewed in Schwarz and Ruhrberg, (2010).

### 3.1 SEMA3 signalling through NRPs in neurons

Several SEMA3 proteins signal through NRPs to regulate neuronal migration, axon guidance or dendrite development (reviewed in Schwarz and Ruhrberg, 2010, Tillo et al., 2012). For example, SEMA3A knockout mouse mutants have defasciculation of cranial and spinal nerves (Taniguchi et al., 1997) and defective path finding of olfactory axons (Cariboni et al., 2011, Schwarting et al., 2000). Agreeing with a role for NRP1 as a SEMA3A receptor in the nervous system, NRP1-null mice phenocopy many neural defects seen in SEMA3A-null mice (e.g. Kawasaki et al., 2002). Whilst most neural SEMA3A responses involve repulsion, increased intracellular cyclic GMP levels can convert repulsive effects into attractive ones, as observed for cortical apical dendrites (Polleux et al., 2000). Even though NRP1 acts mainly as a SEMA3A receptor, and NRP2 as a SEMA3F receptor in neurons, NRP2 can compensate for NRP1 as a SEMA3A-receptor during glioma cell migration *in vitro* and in vomeronasal axon guidance in mouse embryos *in vivo* (Cariboni et al., 2011).

Whilst SEMA3A is generally thought to be downregulated after development is complete, its expression increases locally after injury in mouse models of peripheral nerve injury, where it repels regenerating axons and thereby inhibits the rewiring of the damaged area (Pasterkamp and Verhaagen, 2001). Moreover, inhibiting SEMA3A-dependent signalling with an allosteric competitor of SEMA3A for NRP1 binding in the injured spinal cord creates a more permissive environment for axonal regeneration *in vivo* by promoting angiogenesis, Schwann cell migration and the Schwann cell-mediated myelination of regenerating neuronal fibers (Kaneko et al., 2006).

In the developing *Xenopus* eye, SEMA3A is expressed in the lens and photoreceptors, SEMA3D in the proliferative ciliary marginal zone of the peripheral retina and SEMA3F in neuronal cells of the inner nuclear layer, whilst NRP1 localises to retinal ganglion cell (RGC) axons and dendrites (Kita et al., 2013). Expressing dominant-negative NRP1 or PLXNA1 mutants causes dendrites to extend in various directions from the RGC cell body and therefore prevents the polarised extension of dendrites towards the inner nuclear layer, in the opposite direction of axon extension (Kita et al., 2013). SEMA3A is also upregulated during optic nerve injury, and downregulating its expression may provide a therapeutic strategy to treat this condition, as decreasing SEMA3A expression promotes axon outgrowth in RGCs and reduces apoptosis (Han et al., 2015a, Rosenzweig et al., 2010, Shirvan et al., 2002). The pathological upregulation of SEMA3A in RGCs is also observed in ischemic eye disease, where it promotes the formation of vascular malformations (see below, Sections 3.3, 6.2, 6.3).

### 3.2 SEMA3A signalling through NRPs in the vasculature

SEMA3A, SEMA3C and SEMA3E have been implicated as modulators of the developing vasculature. SEMA3E affects blood vessels independently of NRPs by signalling through PLXND1 (Gu et al., 2005) and will therefore not be a focus of this review. SEMA3C signals through NRP1/PLXND1 complexes during cardiac outflow tract remodelling (Plein et al., 2015a), but this also will not be discussed further. SEMA3A was originally thought to modulate vascular development in the embryo (Serini et al., 2003), but subsequent analyses showed that SEMA3A knockout mice lack obvious defects in developmental blood vessel growth (Bouvree et al., 2012, Vieira et al., 2007). In agreement, loss of semaphorin binding to NRP1 does not affect angiogenesis in mouse embryos, even if they additionally lack NRP2 (Gu et al., 2005, Vieira et al., 2007). Yet, SEMA3A signals through NRP1 to control the development of lymphatic valves (Bouvree et al., 2012, Ochsenbein et al., 2014). Moreover, the in utero delivery of antibodies that inhibit SEMA3A binding to NRP1 causes abnormal lymphatic vessel and valve morphology (Jurisic et al., 2012). The main role for SEMA3A in the vasculature, however, appears to be the modulation of vascular pathology: Firstly, SEMA3A signals through NRP1 to promote pathological angiogenesis in the eye (Joyal et al., 2011) (see Section 2.2.1). Secondly, SEMA3A signals through NRP1 or NRP2 to induce vascular hyperpermeability in the eye and other tissues (Acevedo et al., 2008, Cerani et al., 2013, Hou et al., 2015) (see Sections 6.2, 6.3).

### 3.3 SEMA3A in rodent models of neovascular eye pathology

RGCs express SEMA3A during the hyperoxic phase in the OIR model, and this SEMA3A source repels sprouting vessels away from the most severely ischemic areas after return to normoxia, thus misdirecting them into the vitreous and causing tuft formation (Joyal et al., 2011). The release of interleukin 1 beta from microglia, the resident myeloid population of the retina is responsible for the induction of SEMA3A expression by RGCs (Rivera et al., 2013). Agreeing with an important role for SEMA3A in neovascular tuft formation, administering exogenous SEMA3A into the vitreous reduces pathological neovascularisation in the OIR model (Yu et al., 2013). SEMA3A has also been shown to inhibit VEGF164-induced angiogenesis in the chick chorioallantoic model of pathological vessel growth (Acevedo et al., 2008), raising the possibility that the effect of SEMA3A on blocking neovascular tuft formation is two-fold, firstly by redirecting neovessels away from the vitreous, and secondly by inhibiting VEGF-induced neovessel growth. Treatment with SEMA3A was also reported to block the formation of neovascular lesions after laser-induced CNV by inhibiting TGFβ signalling in endothelial cells (Bai et al., 2014) (see Sections 5.2, 6.2.1).

### 3.4 SEMA3A signalling through NRPs in vascular permeability

A diverse range of secreted proteins can induce vascular hyperpermeability, which is also referred to as acute vascular permeability (Weis and Cheresh, 2005). Whilst it is a beneficial process in acute injury to deliver clotting factors and antibodies to enable wound healing, vascular hyperpermeability can cause tissue-damaging oedema when it remains unresolved, and it is therefore an unwanted complication in many chronic neovascular diseases, especially in the eye (reviewed by Campochiaro, 2015, Weis and Cheresh, 2005). NRP1 has been implicated as a permeability mediator in several studies. Initially, it was shown that SEMA3A injection into adult mouse skin induces vascular hyperpermeability in a NRP1-dependent mechanism (Acevedo et al., 2008, Hou et al., 2015). Whereas SEMA3A is not highly expressed in the healthy retina of adult mice, SEMA3A expression is upregulated in a mouse model of type 1 diabetes induced by streptozotocin treatment, mostly via secretion from RGCs (Cerani et al., 2013). As observed in the skin, SEMA3A-induced retinal vascular leak in diabetic mice was NRP1-dependent (Cerani et al., 2013). SEMA3A is also upregulated during the early hyperglycaemic phase of diabetes in humans, raising the possibility that it presents a therapeutic target to stem excessive vascular permeability in DME (Cerani et al., 2013). SEMA3A also induces brain endothelial vascular leak when injected stereotactically into the cerebral cortex and in models of brain ischemia (Hou et al., 2015). However, using cultured brain endothelial cells, this study found SEMA3A to act via NRP2 and VEGFR1, independently of NRP1, whereby VEGFR1 interacts with the actin regulator MICAL2 to alter cerebro-endothelial cell morphology and permeability (Hou et al., 2015). The blockade of SEMA3A signalling may therefore help to suppress damaging vascular leak in diverse ischemic diseases (see below, Section 6.2).

## 4 Functional significance of VEGF signalling through NRPs

VEGF is essential for the assembly of blood vessels from single cell precursors and the expansion of vessel networks through angiogenesis (reviewed in Ruhrberg, 2003). Accordingly, VEGF is essential also for retinal vascular development (Haigh et al., 2003) and drives neoangiogenesis and/or macular oedema in eye pathologies such as PDR, neovascular AMD and BRVO (Nissen et al., 1998, Saint-Geniez and D'Amore, 2004, Mitry et al., 2013). VEGF also acts on several non-endothelial cell types, including neurons (reviewed in Mackenzie and Ruhrberg, 2012). Finally, VEGF plays a dual role in skin cancer by stimulating angiogenesis through a paracrine mechanism by signalling via VEGFR2 and by promoting cancer stem cell renewal through an autocrine, NRP1-dependent mechanism (Beck et al., 2011). The complexity and versatility of VEGF signalling is enabled by VEGF's expression in several isoforms and their binding to several different receptors, which either act independently of NRP1 or act in co-receptor complexes with NRP1, as described in the following paragraphs.

### 4.1 Alternative splicing of VEGF-A isoforms regulates both receptor and ECM interactions

The presence or absence of the exon 6- and/or 7-encoded protein domains in the VEGF189 and VEGF165 versus VEGF121 isoforms has important functional consequences. These domains promote NRP binding (see section 1.1) but additionally confer a high affinity for heparin *in vitro* that likely reflects an ability to bind heparan sulphate proteoglycans (HSPG) in the extracellular matrix (ECM) and on the cell surface. In agreement with this idea, VEGF189 has a high affinity for ECM and is therefore poorly diffusible, whilst VEGF121 is the most diffusible VEGF-A isoform and VEGF165 has intermediate properties (Houck et al., 1992, Park et al., 1993). Importantly, these differences translate to a differential ability of the VEGF isoforms to be retained in the ECM *in vivo*, with mice expressing the VEGF120 isoform only at the expense of the other VEGF isoforms being unable to form proper growth factor gradients to direct vascular morphogenesis in the brain and retina (Gerhardt et al., 2003, Ruhrberg et al., 2002). In addition to their distinct matrix binding affinities, the VEGF isoforms differ in the spectrum of receptors they bind to (see Section 1.3).

### 4.2 Signalling pathways induced after VEGF binding to receptor tyrosine kinases that interact with NRPs

The VEGF121, VEGF165 and VEGF189 isoforms bind to and activate two tyrosine kinase receptors, VEGFR1 and VEGFR2, with VEGFR2 being the main receptor that conveys VEGF signals in endothelial cells (Koch et al., 2011).

VEGF binding to VEGFR2 induces receptor homodimerisation and oligodimerisation, which promotes autophosphorylation on several tyrosine residues (Y), most notably 951, 1054, 1175 and 1214, and these residues then bind intracellular adaptor proteins to initiate signal transduction (Koch et al., 2011). For example, the recruitment of SCK and GRB2 adaptor proteins to phosphorylated VEGFR2 Y1175 enables VEGF-induced ERK1/2 signalling. The role of NRP1 in modulating VEGFR2 signalling is discussed in detail below (Section 4.4). VEGF165b has been shown to inhibit VEGF165-induced VEGFR2 phosphorylation by acting as a competitive antagonist for VEGF165 in VEGFR2 binding (Woolard et al., 2004).

In contrast to VEGFR2, VEGFR1 is thought to function mainly as a decoy receptor in angiogenesis by trapping VEGF to prevent VEGF binding to VEGFR2 and therefore inhibiting VEGFR2 signalling (Koch et al., 2011, Rahimi et al., 2000). In support of this idea, the tamoxifen-inducible knockout of FLT1 in neonatal mice causes overgrowth of the retinal vasculature, presumably in part by increasing VEGFR2 levels and in part by increasing VEGF availability for VEGFR2 activation (Ho et al., 2012). In contrast, the interaction of VEGFR1 with NRP1 appears to be important for VEGFB signalling, because VEGFB treatment stimulates NRP1-mediated myocardial angiogenesis and arteriogenesis in ischemic pig myocardium through VEGFR1 and not VEGFR2 (Lahteenvuo et al., 2009).

In agreement with the observation that NRP2 interacts with VEGF receptor tyrosine kinases, NRP2 loss reduces both VEGF- and VEGFC-induced cell survival and migration in human microvascular endothelial cells (Favier et al., 2006). *In vivo*, the interaction between NRP2 and VEGFR2 is important for developmental lymphangiogenesis (Xu et al., 2010), tumour-associated pathological lymphangiogenesis (Han et al., 2015b) and likely also for corneal inflammatory neovascularisation-associated lymphangiogenesis (Tang et al., 2015).

### 4.3 Role of NRP1 in modulating VEGF-dependent neural signalling pathways

VEGF164 signalling through NRP1 has been identified in three different types of neurons, facial branchiomotor neurons in the hindbrain to regulate their cell body migration, gonadotropin-releasing hormone (GnRH) neurons to ensure their neuroprotection during their migration from the nasal placode into the brain, and RGC neurons in the retina to enable their axon guidance (reviewed in Mackenzie and Ruhrberg, 2012). In particular, VEGF164 signalling through NRP1 enables the sorting of RGC axons into the contralateral brain hemisphere at the optic chiasm, a major diencephalic brain commissure (Erskine et al., 2011). Interestingly, these VEGF164/NRP1-dependent processes do not require VEGFR2 or VEGFR1, and the NRP1 cytoplasmic domain does not appear to be required for these processes either, suggesting that NRP1 associates with unidentified co-receptors in these cells. VEGF signalling also promotes the survival of RGC neurons in the adult, but this is likely mediated by VEGFR2 independently of NRP1, as VEGF120 with a low affinity for NRP1 is able to activate survival signalling (Nishijima et al., 2007). Interestingly, conditional knockout studies suggest that VEGFR2 signalling in RGCs is not required for retinal morphogenesis (Okabe et al., 2014), while VEGFR2 is instead required for the survival of Muller glia, which are in turn essential for the viability of adult retinal neurons in a model of diabetic retinopathy (Fu et al. Diabetes 2015). Analogous conditional knockout studies will be required to address whether VEGFR2 signalling is also directly required for adult RGC survival in ageing adults.

### 4.4 Role of NRP1 in modulating VEGF-dependent vascular signalling pathways

A role for NRP1 as a co-receptor for VEGFR2 was initially identified through studies in porcine aortic endothelial (PAE) cells; thus, overexpressing NRP1 alongside VEGFR2 increases VEGF165-induced endothelial cell chemotaxis compared to overexpressing VEGFR2 alone (Soker et al., 1998). Moreover, the VEGF165-induced activation of signal transduction cascades involving the ERK1/2 and p38 MAPK kinases was greater in PAE cells co-expressing NRP1 and VEGFR2 compared to cells expressing VEGFR2 alone (Becker et al., 2005). NRP1 alone did not activate these VEGF165-induced pathways (Becker et al., 2005, Soker et al., 1998). Treating PAE cells co-expressing NRP1 and VEGFR2 proteins with a peptide that blocks VEGF165 binding to NRP1 without affecting VEGF165 binding to VEGFR2 also reduced VEGFR2 tyrosine phosphorylation and VEGF165-induced ERK activation, but with a small effect on VEGF165-induced AKT phosphorylation (Jia et al., 2006). Supporting these findings, treating human umbilical vein endothelial cells (HUVECs), which endogenously express both VEGFR2 and NRP1, with anti-NRP1 blocking antibodies reduced VEGFR2 phosphorylation and downstream ERK and AKT activation, although did not abrogate it completely as is instead observed with anti-VEGF antibody treatment (Pan et al., 2007a). In cultured embryoid bodies and in subcutaneous matrigel plugs, VEGF164 also requires NRP1 to activate p38 MAPK kinase, whose inhibition attenuates angiogenesis (Kawamura et al., 2008). In contrast, NRP1 did not promote VEGF121-induced chemotaxis in PAE cells when overexpressed alongside VEGFR2 (Soker et al., 1998). Together, these studies suggest that NRP1 is not essential for VEGFR2-induced signalling, but augments VEGF165-induced, VEGFR2-dependent MAPK signalling.

#### 4.4.1 NRP1 as a regulator of VEGF-dependent arteriogenesis

**Fig. 2:**
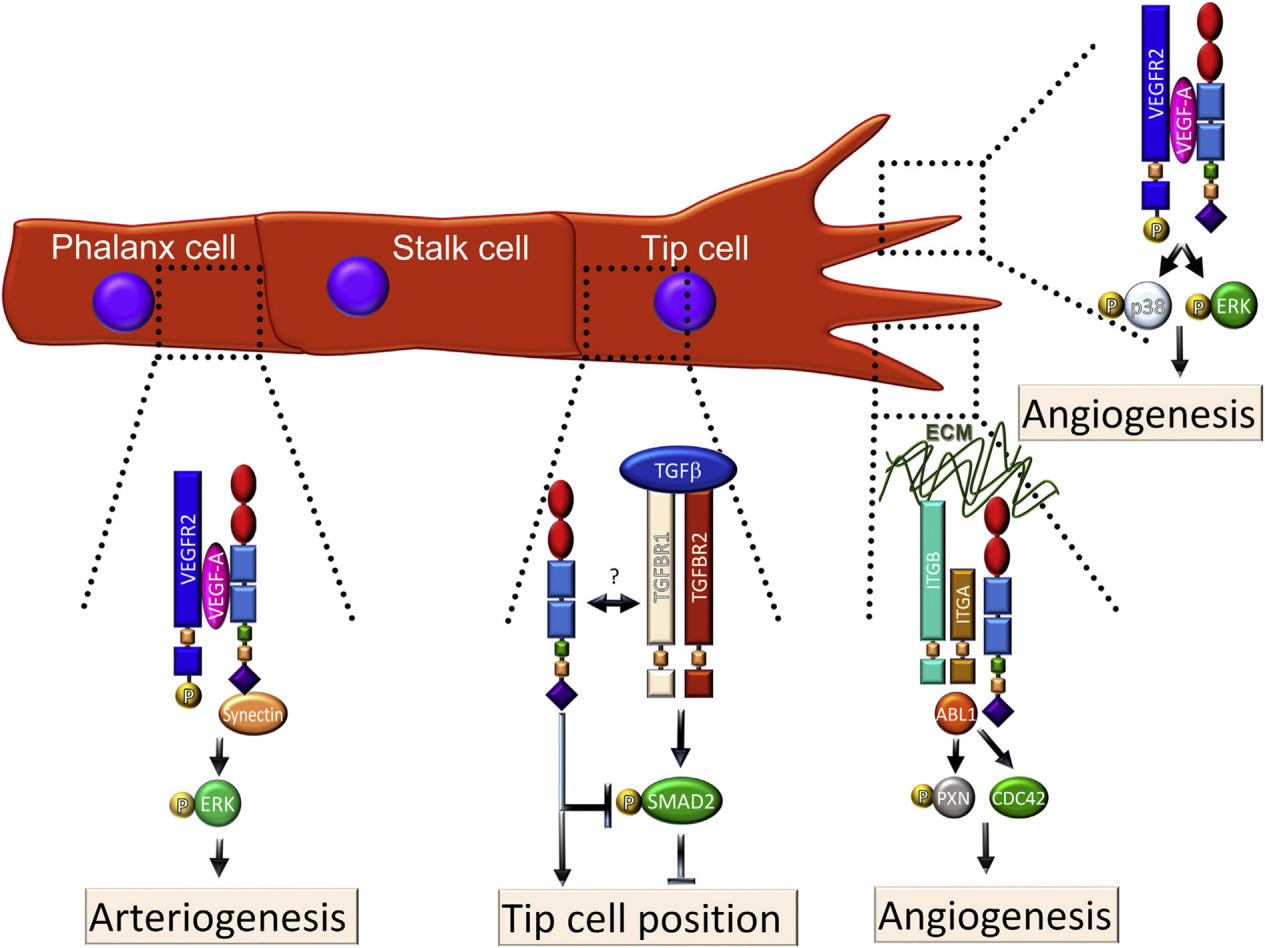
NRP1 regulates multiple signalling pathways in endothelial cells. In tip cells, NRP1 promotes VEGF- and ECM-signalling to promote sprouting angiogenesis. VEGF signalling through VEGFR2/NRP1 complexes induces ERK activation for cell proliferation and migration. Additionally, NRP1 activates ABL1 and CDC42 in response to integrin ligands in the ECM to promote actin remodelling and filopodia formation to enable cell shape changes. NRP1 also suppresses TGF-β signalling to promote tip cell positioning through a mechanism that is incompletely understood. In phalanx cells, NRP1 binds synectin and VEGF and forms a complex with VEGFR2 to enable high level ERK signalling for arteriogenesis.

Arteriogenesis is a process by which small calibre vessels give rise to functional arteries subsequent to increased ﬂow and hence increased shear stress, and this process involves VEGF-induced arterial fate specification, lumen expansion and endothelial cell proliferation (reviewed in Kofler and Simons, 2015). During VEGF165-induced arterial morphogenesis, VEGF165-bound VEGFR2 activates its downstream effectors ERK1/2 in a mechanism that depends on synectin (Lanahan et al., 2010). NRP1 is required to bridge VEGFR2 to synectin, as shown in knock-in mice expressing a mutated NRP1 form lacking the cytoplasmic domain (*Nrp1*^*cyto*/*cyto*^ mice) (Lanahan et al., 2013). Accordingly, VEGF164-stimulated endothelial cells of *Nrp1*^*cyto*/*cyto*^ and synectin-null mice have significantly reduced ERK activation, and both types of mutants show impaired arterial branching in the developing heart, kidney and hindlimb and reduced post-ischemic arteriogenesis in the hindlimb ischemia model (Lanahan et al., 2010, Lanahan et al., 2013). Mechanistically, the NRP1 cytoplasmic tail and synectin link VEGFR2 to a myosin VI-mediated transport machinery that promotes VEGFR2 trafficking into RAB5-positive endosomes, in which VEGFR2 is protected from PTP1b-mediated dephosphorylation, and this enables sustained ERK activation (Lanahan et al., 2013). Together, these data suggest that VEGF164 bridges VEGFR2 to NRP1, which then binds synectin via its cytoplasmic tail to ensure high level ERK activation for arteriogenesis (Fig. 2).

NRP1 was also shown to promote arterial differentiation through studies in mice carrying knockin mutations that abrogate VEGF164 binding to the NRP1 b1 domain, termed *Nrp1*^*Y297A*/*Y297A*^ and *Nrp1*^*D320K*/D*320K*^ mice (Fantin et al., 2014, Gelfand et al., 2014). Both types of mutants have impaired arterial differentiation, with fewer arteries and impaired arterial smooth muscle differentiation in the retina (Fantin et al., 2014, Gelfand et al., 2014). These mutants also have reduced post-ischemic arteriogenesis in the hindlimb ischemia model (Gelfand et al., 2014), similar to mice lacking the NRP1 cytoplasmic domain (Lanahan et al., 2013).

Despite these functional similarities, there are important differences between the two strains of mice with defective VEGF binding to NRP1. Thus, the insertion of a mutated cDNA into the endogenous *Nrp1* locus reduces overall NRP1 levels in *Nrp1*^*Y297A*^ knockin mutants, in addition to impairing VEGF binding to the NRP1 b1 domain (Fantin et al., 2014). The combination of these defects results in a more severe vascular phenotype than in the subsequently generated *Nrp1*^*D320K*^ knockin mutants, which lack VEGF-binding to NRP1 without lowering overall NRP1 levels (Gelfand et al., 2014). Thus, *Nrp1*^*Y297A*/*Y297A*^ mutants have subtle embryonic brain vascular defects that are not observed in *Nrp1*^*D320K*/*​D320K*^ mutants (Fantin et al., 2014, Gelfand et al., 2014). Accordingly, *Nrp1*^*D320K*^ knockin mice with their normal NRP1 expression levels represent a more accurate tool to specifically assess the role of VEGF binding to NRP1.

The hearts of *Nrp1*^*Y297A*/*Y297A*^ mice also have fewer coronary arteries and capillaries, similar to *Vegfa*^*120*/*120*^ mice expressing VEGF120 only at the expense of the heparin/NRP1-binding VEGF isoforms; moreover, a small proportion of these *Nrp1* mutants die perinatally, similar to *Vegfa*^*120*/*120*^ mice that are known to suffer from ischemic cardiomyopathy (Carmeliet et al., 1999, Fantin et al., 2014). Whether heart vascularisation is also defective in *Nrp1*^*D320K*/D*320K*^ mutants has not yet been examined.

VEGF-binding to NRP1 and the NRP1 cytoplasmic domain are also required to promote the spatial separation of retinal arteries and veins. In both *Nrp1*^*Y297A*/*Y297A*^ and *Nrp1*^*cyto*/*cyto*^ retinas of mice on a C57/Bl6 background, arteries and veins cross each other at an abnormally high frequency (Fantin et al., 2011, Fantin et al., 2014), as previously reported for mice with haploinsufficient expression of VEGF in neural progenitors (Haigh et al., 2003). At these crossing sites, the artery is positioned anteriorly to the vein, and both vessels are embedded in a shared collagen sleeve (Fantin et al., 2011). This morphology is similar to the arteriovenous crossings in human eyes that were found to be risk factors for BRVO (see above). *Nrp1*^*Y297A*/*Y297A*^ and *Nrp1*^*cyto*/*cyto*^ mice may therefore provide a suitable genetic model to study the aetiology of BRVO. It has not yet been examined whether the *Nrp1*^*D320K*/D*320K*^ mutants also have an increased frequency of arterio-venous crossing sites.

Taken together, the above studies in mouse models suggest that NRP1 is a key regulator of VEGF-dependent arterial morphogenesis in the developing heart, kidney, hindlimb and eye, and that it also promotes arteriogenesis in adults.

#### 4.4.2 NRP1 as a regulator of VEGF-dependent angiogenesis

The tissue culture work described above had suggested that chemotactic VEGF164 signalling through VEGFR2 and therefore endothelial cell migration are augmented by NRP1. To understand whether these pathways are important for angiogenesis *in vivo*, much work has been carried out in several developmental model systems. This work initially showed that, during angiogenesis, endothelial cells sprout from pre-existing vasculature, whereby the sprouts are composed of stalk cells and led by highly migratory endothelial cells, termed tip cells (Gerhardt et al., 2003, Ruhrberg et al., 2002) (Fig. 2). Tip cells extend filopodia into the extracellular environment and are highly responsive to VEGF, presumably because they express high levels of VEGFR2 (Gerhardt et al., 2003) and NRP1 (Fantin et al., 2013a). The importance of NRP1 for tip-cell led developmental angiogenesis was subsequently characterised in the zebrafish larval trunk (Bovenkamp et al., 2004, Fantin et al., 2015, Lee et al., 2002, Martyn and Schulte-Merker, 2004, Yu et al., 2004), the mouse embryo hindbrain (Fantin et al., 2015, Gerhardt et al., 2004) and the mouse postnatal retina (Aspalter et al., 2015, Fantin et al., 2011, Fantin et al., 2015, Raimondi et al., 2014). Even though all 3 models revealed an essential role for NRP1 in angiogenesis, only retinal angiogenesis has so far been shown to require VEGF binding to NRP1 (Fantin et al., 2014, Gelfand et al., 2014). Supporting the idea that NRP1 is important for ocular vessel growth, the treatment of mice with antibodies that block VEGF binding to NRP1 impairs retinal angiogenesis (Pan et al., 2007a).

**Fig. 3:**
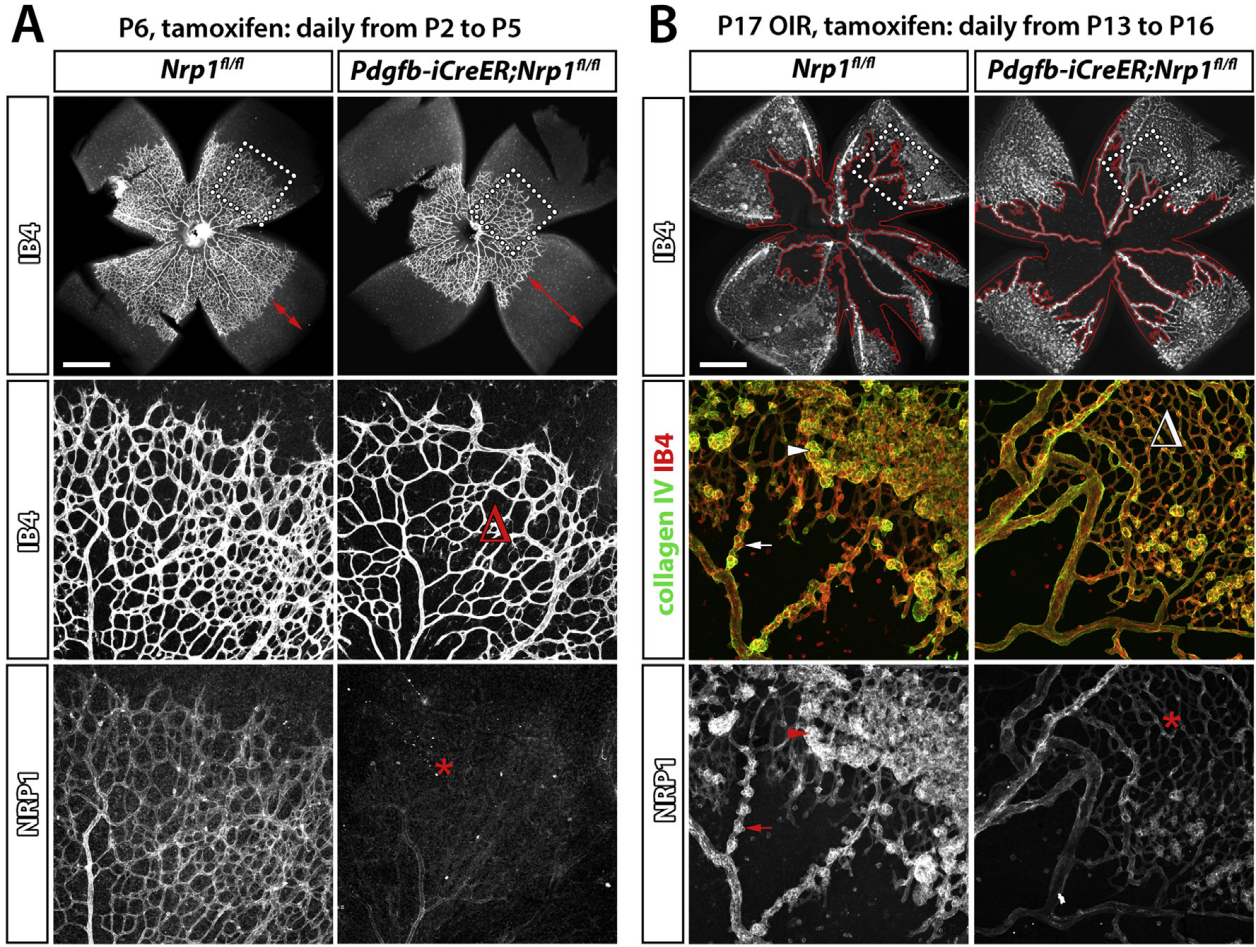
NRP1 promotes physiological and pathological angiogenesis in the retina. (A) P6 retinal vasculature in *Pdgfb-iCre*^*ERT2*^;*Nrp1*^*fl/fl*^ mice and control mice lacking Cre after tamoxifen injection from P2 to P5 and immunolabelling for NRP1 together with IB4. Red arrows indicate the larger distance from the vascular front to the retinal periphery in *Pdgfb-iCre*^*ERT2*^;*Nrp1*^*fl/fl*^ mutants (upper panels). Higher magnifications of the vascular front areas indicated by boxes in the upper panels are shown in the middle panels; Δ indicates reduced lateral connections in the vasculature of mutants. The bottom panels show reduced NRP1 expression in the retinal vasculature of *Pdgfb-iCre*^*ERT2*^;*Nrp1*^*fl/fl*^ mutants compared to controls (asterisks), confirming efficient gene deletion. (B) P17 retinas from *Pdgfb-iCre*^*ERT2*^;*Nrp1*^*fl/fl*^ mice and control littermates lacking Cre in the OIR model after tamoxifen injection from P13 to P16 after return from hyperoxia to normoxia; retinas were immunolabelling for NRP1 and collagen IV together with IB4. The avascular area is outlined in red in the low magnification images in the upper panels, which show the IB4 staining only. Higher magnification of the boxed areas are shown in the middle panels with IB4 together with collagen IV staining to illustrate reduced neovascular tuft formation in *Pdgfb-iCre*^*ERT2*^;*Nrp1*^*fl/fl*^ mutants compared to control. The bottom panels show reduced NRP1 expression in the retinal vasculature of *Pdgfb-iCre*^*ERT2*^;*Nrp1*^*fl/fl*^ mutants. Scale bars: 1 mm (A,B).

To define roles for NRP1 in retinal angiogenesis, two genetic tools have been employed that circumvent the embryonic lethality of full *Nrp1* knockout mice; tamoxifen-inducible, endothelial NRP1 knockout mice (Aspalter et al., 2015, Fantin et al., 2015, Raimondi et al., 2014) and mice with point mutations that impair VEGF binding to NRP1 (see above, section 1.3) (Fantin et al., 2014, Gelfand et al., 2014). To generate inducible, endothelial NRP1 knockouts for retinal angiogenesis studies, mice with conditional *Nrp1*-null (floxed) alleles (*Nrp1*^*fl*/*fl*^) were crossed to mice expressing a tamoxifen-inducible *Cre* transgene under the control of the endothelial *Pdgfb* promoter (*Pdgfb*-*iCre*^*ER*^-*Egfp* (Fantin et al., 2015, Raimondi et al., 2014). Tamoxifen-treated littermate mice expressing or lacking *Cre* from P2 to P5 showed that NRP1 loss impairs the radial extension of the superficial vascular plexus and decreases the vascular network density (Fig. 3A) (Fantin et al., 2015, Raimondi et al., 2014). Furthermore, these studies showed that NRP1 loss severely reduces the number of morphologically identifiable tip cells, vessel sprouts at the vascular front and lateral connections between sprouting vessels (Fantin et al., 2015, Raimondi et al., 2014). Defective radial outgrowth of the retinal vasculature was also seen in mice lacking VEGF binding to NRP1 (Fantin et al., 2014, Fantin et al., 2015, Gelfand et al., 2014). It is likely that these VEGF-dependent roles of NRP1 operate in tip cells and involve the ERK and p38 pathways previously identified in embryoid body and tissue culture models (Fig. 2). However, with endothelial ERK1/2 mutants and p38-mutants not yet analysed, formal proof of this role is still lacking.

In contrast to radial vascular outgrowth, vascular network density was not affected in mice lacking VEGF binding to NRP1 (Fantin et al., 2014, Fantin et al., 2015, Gelfand et al., 2014). The sparse vascular plexus in tamoxifen-inducible endothelial NRP1 knockout mice was better explained by the loss of ECM signalling via NRP1 (see section 5.1) (Fantin et al., 2015). Thus, the important role for NRP1 in angiogenesis is only partly explained by its ability to bind VEGF164. The multiple VEGF-independent roles for NRP1 in retinal angiogenesis are discussed in Section 5.

### 4.5 NRP1 as a regulator of VEGF-induced hyperpermeability

VEGF was originally discovered as a tumour-secreted vascular permeability factor due to its ability to induce the accumulation of ascites (Senger et al., 1983). In particular, VEGF opens the endothelial barrier that normally prevents plasma extravasation into the tissue surrounding the blood vessel. Because VEGF regulates vascular permeability in addition to promoting angiogenic and arteriogenic blood vessel growth, the therapeutic potential to restore blood flow in ischemic tissues is counterbalanced by a risk of hyperpermeability that might cause tissue injury and oedema. Research elucidating the molecular mechanism that steers VEGF-induced responses of vascular endothelial cells towards angiogenesis versus vascular permeability might therefore identify targets suitable for the selective manipulation of VEGF responses in vascular eye disease.

Even though some studies implicated VEGFR2 as the main VEGF receptor that conveys endothelial permeability signalling (Bates and Harper, 2002, Murohara et al., 1998, Sun et al., 2012), several other lines of evidence suggest that NRP1 also contributes to VEGF-induced vascular hyperpermeability, albeit through an incompletely understood mechanism. Initially, *in vitro* experiments showed that the transendothelial electrical resistance (TEER) of PAE cells co-transfected with NRP1 and VEGFR2 decreased after VEGF165 stimulation, whereas it was unaltered in PAE cells transfected with NRP1 or VEGFR2 alone (Becker et al., 2005). As a low TEER indicates a propensity for vascular leak, these experiments raised the possibility that neither VEGFR2 nor NRP1 alone are sufficient to induce vascular leakage. However, these experiments were conducted in cells that do not have endogenous VEGF receptors and therefore lack essential characteristics of true endothelial cells. It is therefore interesting that function-blocking antibodies for NRP1 inhibit VEGF165-induced permeability in primary pulmonary endothelial cells, whilst function-blocking antibodies for VEGFR2 did not affect permeability, even though they abolished VEGF-induced chemotaxis (Becker et al., 2005).

Whilst some *in vivo* studies support the idea that NRP1 has a central role in VEGF165-induced vascular permeability, others found it not to be important. Thus, one study reported that the genetic deletion of NRP1 in endothelial cells impairs skin permeability after intradermal injection of SEMA3A or VEGF164 (Acevedo et al., 2008). However, another study reported normal VEGF165-induced vascular permeability in the retina of endothelial NRP1 knockout mice, even though these mice had defective SEMA3A-induced permeability (Cerani et al., 2013). These observations raise the possibility that the NRP1 contribution to VEGF-induced permeability is context dependent.

Opposing results have also been reported in studies testing the effect of NRP1-blocking antibodies on VEGF-induced permeability. Two studies using the same NRP1-blocking antibody showed that antibody treatment has no effect on VEGF164-induced vascular permeability in mouse skin (Acevedo et al., 2008, Pan et al., 2007a), whereas a different NRP1-blocking antibody inhibited VEGF164-mediated permeability in the mouse skin (Teesalu et al., 2009). It is not known whether these opposing results can be explained by the different effectiveness of function-blocking antibodies in reducing NRP1-mediated vascular permeability depending on the NRP1 domain they bind to or the specific experimental context they are used in.

In agreement with a NRP1 role in VEGF-induced permeability, a heptapeptide that blocks VEGF binding to NRP1 (see Section 6.1.2) attenuates both neovascularisation and vascular permeability in a mouse model of diabetic retinopathy (Wang et al., 2015a). Moreover, soluble NRP1 expression in mouse skin strongly reduces vascular leakage induced by intradermal VEGF injection (Mamluk et al., 2005), perhaps because it acts as a VEGF164 trap.

## 5 VEGF- and SEMA3A-independent NRP1 signalling pathways in the vasculature

Constitutive NRP1 knockout mice are embryonically lethal with vascular defects in the yolk sac, spinal cord and brain (Fantin et al., 2013a, Gerhardt et al., 2004, Jones et al., 2008, Kawasaki et al., 1999), and they also have defective remodelling of the large vessels of the heart (Kawasaki et al., 1999, Plein et al., 2015a). However, NRP1 function in angiogenesis has been best studied in the mouse embryo hindbrain and led to the demonstration that NRP1 is essential for endothelial tip cell function and that it promotes angiogenesis in VEGF-independent pathways (Fantin et al., 2014, Fantin et al., 2015, Fantin et al., 2013a, Gerhardt et al., 2004). In the mouse, vessels sprout from the perineural vascular plexus into the hindbrain at E9.5 and then grow towards the ventricular zone, attracted by VEGF secreted from neural progenitors (Breier et al., 1992, Haigh et al., 2003, Ruhrberg et al., 2002). From E10 onwards, radial vessels extend sprouts at near right angles to extend beneath the ventricular hindbrain surface, forming the subventricular vascular plexus (SVP) following anastomosis of neighbouring sprouts (Fantin et al., 2010). Whilst the SVP begins to form from E10.5 onwards in wildtype mice, heterozygous *Nrp1*-null hindbrains have a less dense SVP, and homozygous *Nrp1*-null hindbrains lack an SVP at this stage entirely (Fantin et al., 2015). Thus, NRP1 promotes hindbrain vascularisation in a dose-dependent manner.

Whereas vascular ingression largely recovers in heterozygous *Nrp1* mutant hindbrains, the radial vessels that enter homozygous *Nrp1*-null hindbrains terminate in dead-ended vessel tufts rather than a branched network (Fantin et al., 2015, Gerhardt et al., 2004). This defect is phenocopied in mouse mutants lacking NRP1 specifically in endothelium, whereas NRP1 expression by neural progenitors and tissue macrophages in the vascular environment is dispensable for hindbrain vascularisation (Fantin et al., 2013a). Recent genetic mosaic analyses of hindbrain blood vessels demonstrated a key role for NRP1 in endothelial tip cells by showing that NRP1 loss prevents endothelial cells from attaining the tip position (Fantin et al., 2013a).

Although it was originally thought that vascular defects in NRP1-deficient mice were caused by impaired VEGF signalling through endothelial NRP1 (e.g. Gu et al., 2003), mice with the knock-in Y297A mutation that abrogates VEGF binding to NRP1 (Herzog et al., 2011) have only a mild vascular phenotype in the hindbrain vasculature (Fantin et al., 2014). Moreover, this mild defect was distinct to the severe phenotype of full NRP1 knockouts and likely due to a reduction in overall NRP1 levels caused by the mutant allele (Fantin et al., 2014), rather than defective VEGF binding to NRP1, as subsequent work with mice carrying a D320K mutation that does not affect NRP1 expression showed normal brain angiogenesis in the absence of VEGF binding to NRP1 (Gelfand et al., 2014). NRP1 can therefore promote angiogenesis by regulating VEGF-independent pathways, and these pathways, whilst particularly important for embryonic angiogenesis, likely also contribute to postnatal angiogenesis, as discussed in the following sections.

### 5.1 NRP1 as a modulator of extracellular matrix signalling in the vasculature

Several studies have identified functional interactions of NRP1 with integrin-mediated pathways. Human endothelial cells *in vitro* require NRP1 for integrin-mediated adhesion to low concentrations of integrin ligands such as fibronectin (Murga et al., 2005, Valdembri et al., 2009). Moreover, NRP1 promotes fibronectin fibrillogenesis in arterial endothelial cells *in vitro* by regulating intracellular trafficking of activated α5β1 in a mechanism that requires the NRP1 cytoplasmic domain (Valdembri et al., 2009). This pathway was originally proposed to modulate angiogenesis. However, the NRP1 cytoplasmic domain is not required for angiogenesis, but instead promotes arteriogenesis *in vivo* (Fantin et al., 2011, Lanahan et al., 2013). Further work is therefore required to establish whether NRP1-modulated, integrin-driven fibronectin assembly is important for arteriogenesis. NRP1 also facilitates fibronectin fibril assembly in the tumour microenvironment, promoting desmoplasia (Yaqoob et al., 2012). In this process, the NRP1 cytoplasmic domain was shown to recruit the intracellular kinase ABL1 (Yaqoob et al., 2012), a known integrin interactor (Lewis et al., 1996). Together, these observations suggest that NRP1 plays important roles in integrin-mediated matrix remodelling.

In agreement with an important role for NRP1 in integrin signalling, we recently showed that NRP1 promotes endothelial cell migration in response to fibronectin (Fantin et al., 2015, Raimondi et al., 2014). This pathway differed from previously identified NRP1-mediated mechanisms in angiogenesis, because it was functional at fibronectin concentrations that do not challenge endothelial cell adhesion and operates independently of NRP1's conventional role as a co-receptor in the VEGF/VEGFR2 pathway. Instead, this pathway involves the activation of two proteins that promote actin cytoskeleton remodelling, ABL1 (Raimondi et al., 2014) and the small RHO-GTPase CDC42 (Fantin et al., 2015) (Fig. 2).

**Fig. 4:**
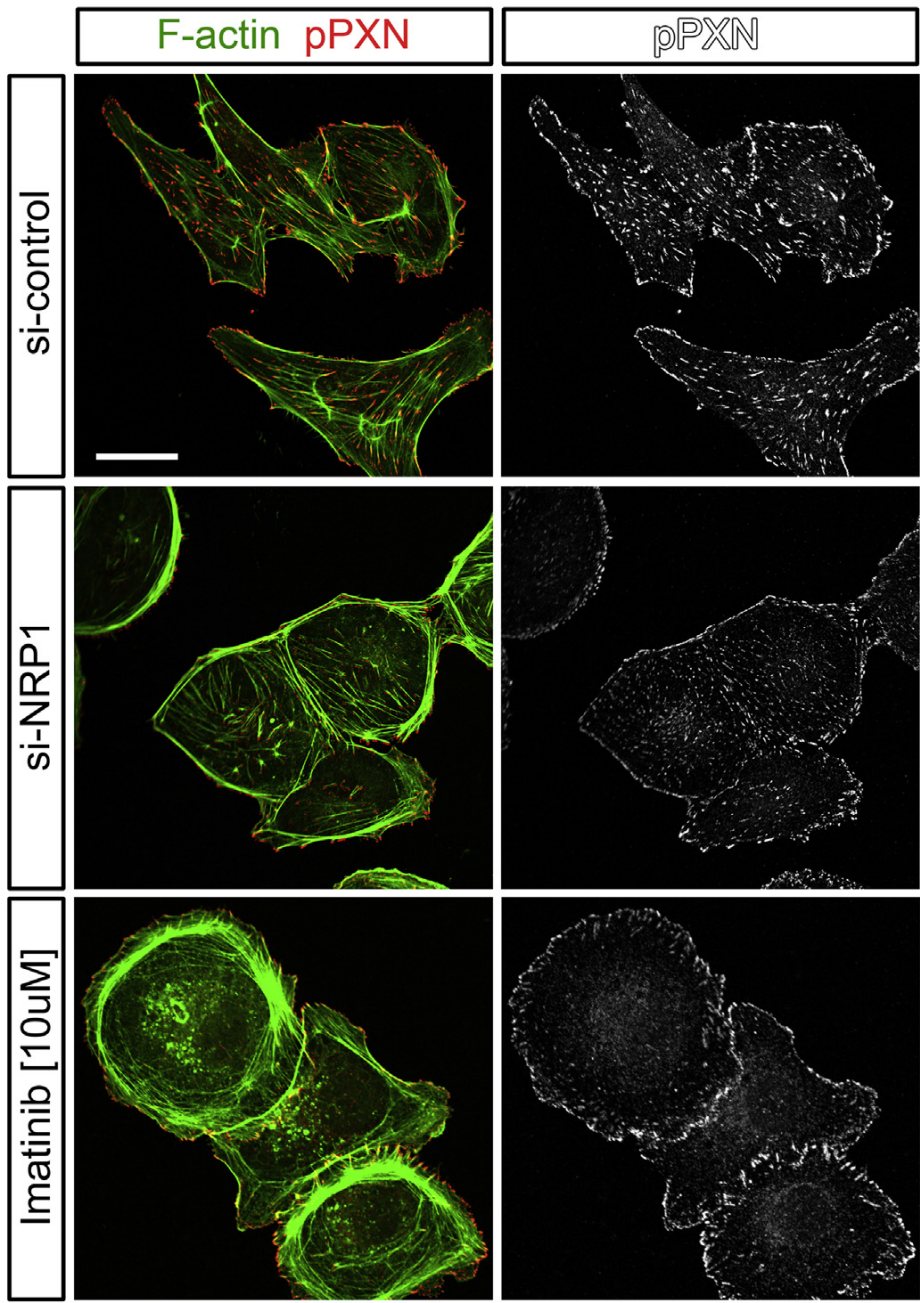
NRP1 downregulation and Imatinib treatment similarly inhibit actin cytoskeleton remodelling and reduce paxillin phosphorylation. HDMEC transiently transfected with non-targeting or NRP1-targeting siRNA or treated with 10 μM Imatinib were seeded on fibronectin for 4 h and immunofluorescently labelled for pPXN Y118 (red) together with the F-actin marker phalloidin (green). The single channel for pPXN staining is shown on the right hand side. Scale bar 20 μm.

Initially, *in vitro* studies with human primary endothelial cells showed that NRP1 forms a complex with ABL1 and stimulates phosphorylation of paxillin in response to fibronectin stimulation (Raimondi et al., 2014) (Fig. 4). As ABL1 is known to phosphorylate paxillin (Lewis and Schwartz, 1998) and paxillin phosphorylation is important for focal adhesion turnover during cell migration (Zaidel-Bar et al., 2007), this study provided the first mechanistic explanation why NRP1 is important for endothelial cell migration. Even though tumour studies had shown that the NRP1 cytoplasmic domain is important for ABL1 function in fibronectin fibrillogenesis, the NRP1 cytoplasmic domain is not required for angiogenesis (Fantin et al., 2011). It is likely that ABL1 kinase can be recruited to NRP1-containing multiprotein complexes through other interactions that may be involve integrins, as NRP1 interacts with integrins (Fukasawa et al., 2007, Robinson et al., 2009), and integrins interact with ABL kinases (Lewis and Schwartz, 1998).

A subsequent study from our lab showed that NRP1-mediated ABL1 signalling is also required for fibronectin-induced activation of CDC42 (Fantin et al., 2015). CDC42 is a small RHO-GTPase that cycles between a GTP-bound active and a GDP-bound inactive state to regulate actin cytoskeleton remodelling, filopodia extension and directional migration in various cell types (Heasman and Ridley, 2008), including endothelial cells (Abraham et al., 2015, Fantin et al., 2015, Wakayama et al., 2015). In particular, NRP1/ABL1-mediated CDC42 activation promotes the extension of filopodia as well as cell shape changes for cell migration in response to ECM stimulation (Fantin et al., 2015). However, the specific mechanism by which ABL1 kinase can enable CDC42 activation has not yet been determined.

ECM-driven, NRP1-dependent actin remodelling promotes both physiological and pathological angiogenesis in the mouse (Fantin et al., 2015, Raimondi et al., 2014). Thus, the primary plexus of the developing retinal vasculature of inducible, endothelial specific NRP1 knockout mice had fewer tip cells and branchpoints (Fig. 3), and this defect was phenocopied in mice treated with the ABL kinase inhibitor Imatinib or the CDC42 inhibitor ML141 (Fantin et al., 2015, Raimondi et al., 2014). Consequently, the primary vascular plexus of mice lacking ECM-mediated NRP1 signalling via ABL1 and CDC42 appeared greatly underdeveloped. In agreement with the inhibitor studies, the genetic deletion of CDC42 in postnatal retinal vessels reduces filopodia formation and leads to aberrant vascular sprouting and remodelling (Barry et al., 2015).

Imatinib-treatment additionally causes a small decrease in vascular extension of the primary plexus across the developing retina (Raimondi et al., 2014). This phenotype is likely explained by reduced fibronectin deposition at the vascular front (Raimondi et al., 2014), because it was recently shown that loss of fibronectin expression in astrocytes causes a small delay in retinal vascular extension (Stenzel et al., 2011). Importantly, NRP1 mutants have a more severe reduction in vascular extension than Imatinib-treated mice, in agreement with an additional role of NRP1 in promoting chemotactic VEGF signalling through VEGFR2 in endothelial tip cells during retinal vascularisation (Fantin et al., 2014, Fantin et al., 2015, Gelfand et al., 2014) (Fig. 2). Thus, NRP1 plays a dual role in angiogenesis by independently promoting ECM-stimulated and growth factor-induced signals in EC in physiological retinal angiogenesis (Fig. 2). Both NRP1 roles are also important for pathological retinal angiogenesis (Section 6).

Despite ample evidence that NRP1 helps to convey extracellular matrix signals by modulating integrin function, the molecular mechanism by which NRP1 interacts with integrins is not yet understood. In particular, it remains to be examined whether NRP1 interacts with integrin β1 directly, or if their interaction is only indirect as part of a multiprotein complex. Moreover, it is not yet clear how the VEGF- and ECM-driven NRP1 pathways described above intersect with NRP1-mediated TGFβ signalling, discussed in the next section.

### 5.2 NRP1 in TGFβ signalling during vascular development

The TGFβ family of secreted cytokines regulates cell proliferation, migration, differentiation and apoptosis. Three TGFβ isoforms exist, produced by the *TGFB1*, *TGFB2* and *TGFB3* genes. Each *TGFB* gene encodes a pro-protein of 50 kDa, which is cleaved by furin enzymes and then dimerises; the cleaved dimer binds to the dimeric pro-protein, also referred to as latency-associated peptide (LAP), through non-covalent interaction. Upon secretion, the LAP/TGFβ complex is covalently linked to the latent TGFβ binding protein (LTBP) via a disulfide bond to maintain TGFβ in an inactive state (Taylor, 2009). Studies in T-cells revealed that NRP1 binds to active TGFβ1, LAP and the latent form of TGFβ that consists of a complex between TGFβ1 and LAP (Glinka and Prud'homme, 2008). It was also shown that VEGF165 competes with LAP and TGFβ1 for NRP1 binding in these cells, suggesting that the NRP1 binding sites for VEGF165 and TGFβ1 overlap (Glinka and Prud'homme, 2008).

NRP1 and NRP2 form a complex with the TGFβ receptors TGFBR1 and TGFBR2, independently of TGFβ1 binding. The TGFBR1 can either be ALK1 and ALK5. Studies in breast cancer cells showed that NRP1 and NRP2 have a similar affinity for TGFBR1, whereas NRP2 has a higher affinity for TGFBR2 that TGFBR1 (Glinka et al., 2011). In these cells and also in cardiomyocytes, NRP1 downregulation reduces TGFβ-dependent phosphorylation of the signal transducer SMAD2, indicating that NRP1 can positively regulate TGFβ signalling (Rizzolio and Tamagnone, 2011). In agreement, loss of NRP1 expression specifically in smooth muscle cells and cardiomyocytes decreased survival in mice and correlated with cardiomyopathy (Wang et al., 2015b).

TGFβ signalling is crucial for early vascular development, with 50% of *Tgfb1*-null mice showing defective yolk sac vasculogenesis and haematopoiesis (Dickson et al., 1995). It has not yet been examined whether NRP1 is involved in regulating these early TGFβ signalling events. In contrast, a role for NRP1 in modulating TGFβ signalling has been identified in postnatal angiogenesis. Thus, NRP1-deficient endothelial cells have increased TGFβ signalling and show hyperphosphorylation of SMAD effectors, which impairs tip cell formation and therefore sprouting angiogenesis in the postnatal retina (Aspalter et al., 2015) (Fig. 2). In particular, VEGF-dependent, but NRP1-independent upregulation of DLL4 in tip cells activates notch signalling in stalk cells, which in turn decreases NRP1 levels in stalk cells, thus relieving the inhibition of SMAD-dependent signalling and thereby promoting stalk cell behaviour (Aspalter et al., 2015). The defective ability of NRP1-deficient cells to reach the tip position can be rescued by the endothelial deletion of one copy of either ALK1 or ALK5 (Aspalter et al., 2015). Also agreeing with a role for NRP1 in suppressing endothelial TGFβ signalling, NRP1 forms an intercellular protein complex with β8 integrin on neuroepithelial cells that promotes cell–cell adhesion between endothelial and neuroepithelial cells and balances TGFβ signalling via SMAD effectors (Hirota et al., 2015). It remains to be investigated why NRP1 has opposing roles in TGFβ signalling in endothelial cells versus smooth muscle or cancer cells.

### 5.3 NRP1 in PDGF signalling in smooth muscle cells

NRP1 can modulate signalling induced by the platelet-derived growth factor PDGFA and PDGFB. As mentioned above, NRP1 interacts with PDGFRs, and NRP1 downregulation reduces PDGF-induced PDGFR phosphorylation and therefore cell migration and proliferation of human mesenchymal stem cells (Ball et al., 2010). NRP1 also forms a constitutive complex with PDGFRα in primary human aortic smooth muscle cells, stimulating PDGFR phosphorylation and the PDGF-dependent phosphorylation of the adapter protein p130Cas (Crk-associated substrate) to increase smooth muscle cell migration (Evans et al., 2011, Pellet-Many et al., 2011). A recent study reported that NRP1 expression in smooth muscle cells does not play major roles in the development or the maturation of the aorta or retinal arteries; instead, NRP1 preserves smooth muscle contractility and motility in the gastrointestinal tract (Yamaji et al., 2015). However, further *in vivo* studies are required to establish whether lack of PDGF signalling is responsible for the defective function of visceral smooth muscle lacking NRP1. It also remains to be investigated whether PDGF enables bridge formation between NRP1 and PDGF receptors to increase signal transduction in a mechanism akin to that induced by VEGF165 during NRP1-VEGFR2 complex formation and signalling (see above).

## 6 NRP1 as a therapeutic target in neovascular eye disease

We recently reported that NRP1 is expressed in the endothelial cells of neovascular lesions in the OIR model (Fig. 3B) (Raimondi et al., 2014). Moreover, we demonstrated that the tamoxifen-inducible, endothelial specific ablation of NRP1 in postnatal mice impaired the revascularisation of vasoobliterated areas and reduced the formation of neovascular lesions in this model (Fig. 3B) (Raimondi et al., 2014). Interestingly, endothelial NRP1 targeting caused a slightly stronger effect than Imatinib treatment, which blocks ABL1 as a downstream effector of NRP1 in ECM signalling (Raimondi et al., 2014). This observation raises the possibility that NRP1 has a dual role promoting ECM-stimulated and VEGF-induced signals in endothelial cells in OIR, as observed during physiological angiogenesis (see above). A dual role for NRP1 in VEGF- and ECM-driven angiogenesis during pathological angiogenesis may also explain why anti-VEGF and anti-NRP1 treatments have an additive effect in reducing tumour growth (Pan et al., 2007a). However, these ideas remain to be tested experimentally.

NRP1 is expressed by both endothelial cells and RPE cells in choroidal neovascular membranes of AMD patients (Lim et al., 2005). Agreeing with a role for NRP1 in neovascular AMD, a single-nucleotide polymorphism (SNP) in *NRP1* (rs2070296) is associated with decreased response to ranibizumab (Lucentis), as assessed by treatment-induced improvement of visual acuity (Lores-Motta et al., 2016). The cause of this association is unlikely to be a change in protein sequence as this variant represents a synonymous mutation at position 537 in the cDNA sequence (http://www.ensembl.org/Homo_sapiens/Transcript/Sequence_cDNA?db=core;g=ENSG00000099250;r=10:33177492-33336262;t=ENST00000374867). Moreover, this SNP does not appear to be in linkage-disequilibrium with any other known coding SNPs (linkage-disequilibrium data from the 1000 Genomes Project). It has not yet been examined whether this SNP is associated with regulatory elements that control gene expression.

The importance of NRP1 in animal models of vascular pathology and tumourigenesis, combined with multiple descriptions of NRP1 expression in human disease, suggests that NRP1 may be a useful therapeutic target for several vessel-associated diseases. NRP1 function may be inhibited with function blocking antibodies, peptides, small molecules or micro RNA (miRNA) mimics. Most studies seeking to target NRP1 function have assessed a candidate agent's ability to modulate endothelial cell phenotypes in commonly used *in vitro* assays and/or examined their ability to inhibit tumour growth and angiogenesis in mouse models. Many of the principle findings obtained through these studies will likely be relevant for ocular disease. The search for NRP1 blocking agents has so far focussed mainly on identifying compounds that inhibit VEGF164 binding to NRP1, but recent studies raised the possibility that VEGF-independent NRP1 functions may also be possible therapeutic targets, such as manipulating SEMA3A signalling through NRP1 or NRP1-mediated ECM signalling via integrins.

### 6.1 Agents that target VEGF binding to NRP1

#### 6.1.1 Monoclonal antibodies that target NRP1

Genentech utilised a synthetic, naïve-antibody phage library to develop NRP1 function-blocking antibodies (Liang et al., 2007). They subsequently characterised two of these agents in detail, anti-NRP1^A^, which targets the NRP1 a1-a2 domains to inhibit SEMA3A binding, and anti-NRP1^B^, which targets the b1-b2 domain to inhibit VEGF164 binding (Pan et al., 2007a). The dissociation constants (K_d_) of anti-NRP1^A^ and anti-NRP1^B^ are 0.9 nM and 0.4 nM for human NRP1, respectively, and both antibodies also bind mouse NRP1 (Pan et al., 2007a). Although anti-NRP1^A^ and anti-NRP1^B^ target distinct NRP1 domains and prevent binding to different ligands, both are capable of inhibiting VEGF164-dependent endothelial cell migration and angiogenic sprouting *in vitro* (Pan et al., 2007a). In fact, each antibody is capable of preventing the formation of the VEGF164-induced NRP1-VEGFR2 complex, thereby explaining why both antagonise endothelial VEGF164 signalling (Pan et al., 2007a). In agreement, both antibodies inhibit corneal neovascularisation in rats and impair retinal vascular remodelling in mice (Pan et al., 2007a). In neonatal mice, the combined anti-VEGF and anti-NRP1^B^ treatment decreases retinal vascular density more than anti-VEGF treatment alone (Pan et al., 2007a). This observation raises the possibility that each reagent alone is either suboptimal at targeting VEGF signalling, or that anti-NRP1^B^, even though designed to target VEGF-dependent pathway, also targets VEGF-independent pathways.

MNRP1685A is a phage-derived monoclonal antibody equivalent to NRP1^B^ that recognises NRP1 in humans as well as mice, rats and monkeys (Xin et al., 2012a) and has been evaluated in human phase I studies of solid tumour growth to define its pharmacokinetics, pharmacodynamics and safety profile (Weekes et al., 2014, Xin et al., 2012b). The synergistic effect of anti-NRP1^B^ and anti-VEGF in mouse models of ocular neovascularisation (Pan et al., 2007a) provided the rationale for a further phase 1b trial of MNRP1685A (Patnaik et al., 2014). This trial assessed the safety of treating patients afflicted by advanced tumours with MNRP1685A together with Bevacizumab, a humanised monoclonal antibody that inhibits VEGF and has been approved for the treatment of several types of cancer. Disappointingly, high rates of proteinuria were observed, exceeding those observed with Bevacizumab alone, suggesting that further clinical trials with the combination therapy were not warranted (Patnaik et al., 2014). It has not yet been investigated whether the combined treatment might instead be effective to treat eye disease, for which the intravitreal delivery of therapeutics would likely reduce the risk of systemic adverse effects such as proteinuria. Moreover, MNRP1685A has not yet been tested for efficacy as a single agent to treat neovascular eye disease.

#### 6.1.2 Peptides targeting NRP1

Several different types of NRP1-targeting peptides are available that differ in their design, but have the common purpose of targeting the VEGF binding domain of NRP1.

##### 6.1.2.1 C-end rule peptides

Phage display screens for inducers of cell internalisation and tissue penetration identified a class of peptides with a R/K/XXR/K C-terminal motif that were capable of binding NRP1 and subsequently termed C-end rule (CendR) peptides (Teesalu et al., 2009). The CendR motif is endogenously present at the C-terminus of VEGF and in several semaphorins after furin cleavage (Parker et al., 2013, Teesalu et al., 2009). The RPARPAR peptide was the first CendR peptide studied in detail, because of its ability to induce NRP1 internalisation and vascular permeability (Teesalu et al., 2009). Different combinations of the central ‘XX’ amino acids of the CendR motif have since been examined, and it was concluded that several combinations are sufficient for NRP1 binding, but that PA, PP or PR residues in the XX position are optimal (Zanuy et al., 2013). The CendR peptides appear to be most effective when assembled into multimers, likely because the *K*_d_ of each individual peptide is low, being in the micromolar range, or because multivalent ligands are required for receptor oligomerisation (Teesalu et al., 2009). Aside from modulating NRP1 function, CendR peptides have been proposed as useful agents for delivering cargo into endothelial and tumour cells, as they can be chemically coupled to other molecules and undergo NRP1-mediated endocytosis (Sugahara et al., 2009).

##### 6.1.2.2 A7R

The heptapeptide ATWLPPR (A7R) was originally thought to antagonise the VEGF interaction with VEGFR2 (Binetruy-Tournaire et al., 2000), but is now known to impair VEGF binding to NRP1 (Starzec et al., 2006). With a C-terminal arginine residue, this sequence resembles a C-end rule peptide, and with prolines in the XX position, this peptide has the correct motif for high NRP1 affinity (Zanuy et al., 2013). The C-terminal LPPR sequence in particular was found to be essential for antagonising the interaction of VEGF with NRP1 (Starzec et al., 2007). A7R reduces endothelial cell proliferation and tube formation in matrigel-based fibroblast co-culture assays *in vitro*. *In vivo*, A7R administration reduces vascular density and tumour size in a breast cancer cell xenograft model, but has no direct effect on cancer cell proliferation (Starzec et al., 2006). The vascular effects of A7R treatment are not limited to angiogenesis. The peptide can prevent VEGF-induced vascular permeability in a mouse model of VEGF-mediated neuroinflammation (Suidan et al., 2012), and, within the context of ocular disease, A7R treatment has recently been suggested to prevent blood-retinal barrier dysfunction in a diabetic mouse model (Wang et al., 2015a).

##### 6.1.2.3 Bicyclic peptides

Bicyclic peptides are polypeptides with two circular units. They tend to have higher specificity for their targets and are more resistant to circulating proteases than linear peptides (Baeriswyl and Heinis, 2013). EG3287 is a bicyclic peptide that is designed upon the sequence encoded by exons 7 and 8 of VEGF and is cyclised through disulfide bonding (Jia et al., 2006). Although cyclised, the folding of EG3287 resembles that of the VEGF C-terminus, as demonstrated by nuclear magnetic resonance (NMR) (Jia et al., 2006). EG3287 antagonises VEGF binding to NRP1 expressed by PAECs with an IC_50_ of 3 μM, but has no antagonistic effect on VEGF binding to VEGFR1- or VEGFR2-expressing PAECs (Cheng et al., 2004). Furthermore, EG3287 prevents the formation of the VEGFR/VEGF/NRP1 complex and VEGFR2 phosphorylation (Jia et al., 2006).

#### 6.1.3 Small molecule inhibitors of NRP1

Small molecule inhibitors can be produced with greater cost-effectiveness and have superior pharmacokinetic properties compared to peptide-based agents. Accordingly, several studies have attempted to identify small molecule inhibitors of the VEGF-NRP1 interaction. The earliest of these endeavours was a commercial screen of a small molecule library that identified 3-amino-1-cyano-indolizines as potent inhibitors of the VEGF-NRP1 interaction. The most potent of these molecules inhibited VEGF-NRP1 binding with an IC_50_ of 2 μM (Bedjeguelal et al., 2006). However, published evidence is not available to determine whether these compounds inhibit VEGF-induced angiogenesis, and it is not known if they may be useful in preclinical or clinical settings.

The VEGF-NRP1 binding antagonist termed EG00229 is a small molecule inhibitor based upon the previously described EG3287 bicyclic peptide (Jarvis et al., 2010). NMR and crystallography data demonstrated that this compound binds the VEGF-binding pocket of NRP1 (Jarvis et al., 2010). EG00229 inhibited VEGF binding to NRP1 in a cell free assay with an IC_50_ of 3 μM and in HUVECs with an IC_50_ of 23 μM, whereby the reduced potency in the cell assay is likely explained by the presence of the alternative VEGF receptors VEGFR1 and VEGFR2, which are not targeted by EG00229 (Jarvis et al., 2010). This compound showed promising results in impairing VEGF-induced migration in HUVECs, and a recent *in vivo* study suggested that it may also be suitable for NRP1 targeting when delivered locally via a minipump (Miyauchi et al., 2016).

The most recent small molecule antagonists of VEGF164 binding to NRP1 were identified using an *in silico* approach, in which a small molecule library was screened for compounds that held a structure likely to fit the VEGF binding pocket of NRP1. Candidates were subsequently validated as inhibitors of VEGF-binding to NRP1 and of HUVEC proliferation (Borriello et al., 2014). The lead candidate N-[5-(1H-benzimidazol-2-yl)-2-methylphenyl]-N’-(2,3-dihydro-1,4-benzodioxin-6-ylcarbonyl)thiourea had a higher potency in inhibiting HUVEC proliferation than EG00229 (IC_50_ = 0.2 μM compared to an IC_50_ = 160 μM for EG00229), even though it was less potent in blocking VEGF binding to NRP1 (IC_50_ = 34 μM compared to IC_50_ = 16 μM for EG00229) (Borriello et al., 2014). Furthermore, this compound had demonstrable anti-angiogenic properties in an *in vitro* tubulogenesis assay and a tumour xenograft model (Borriello et al., 2014). However, the drastic reduction in tubulogenesis or angiogenesis correlated with significant levels of apoptosis in both models, suggesting that this compound might be more suitable for tumour targeting than the treatment of neovascular eye disease.

### 6.2 Targeting VEGF-independent NRP1 pathways

#### 6.2.1 Therapeutic potential of targeting SEMA3A signalling through NRP1

Based on work in the OIR mouse model, it has been proposed that exogenous SEMA3A may be administered to redirect neovascularisation from the vitreous into the retina in retinopathies (Joyal et al., 2011) (see Section 3.3). In effect, this type of therapeutic approach would seek to neutralise the harmful effects of intraretinal endogenous SEMA3A with a counter-gradient of exogenous SEMA3A. However, it has not yet been examined if this strategy induces vascular leak. This is an important consideration, given the finding that SEMA3A induces retinal oedema in a mouse model of PDR and the observed upregulation of SEMA3A in the retina during the early hyperglycaemic phase in diabetic patients with macular oedema (Cerani et al., 2013) (see Sections 3.3, 3.4).

Alternatively, and analogous to a SEMA3A knockout, blockade of endogenous, intraretinal SEMA3A signalling might also prevent repulsion of vessels from the neuroretina into the vitreous and additionally prevent oedema, for example in diabetic macular oedema. As SEMA3A does not play any known role in vascular maintenance, this strategy may not suffer from impaired choriocapillaris maintenance, as may be the case for long-term anti-VEGF therapy. Moreover, SEMA3A is pro-apoptotic for endothelial cells *in vitro* (Guttmann-Raviv et al., 2007, Klagsbrun and Eichmann, 2005), raising the possibility that SEMA3A blockade might also promote endothelial survival in diseases with vascular drop-put, such as diabetic retinopathy. In addition to providing signals to endothelial cells in vascular eye pathology, SEMA3A was also hypothesised to promote the recruitment of myeloid cells as well as myeloid cell-mediated pathological neovascularisation in the OIR model (Dejda et al., 2014). However, the relative importance of SEMA3A and VEGF164 for the attraction and function of NRP1-expressing myeloid cells was not functionally distinguished in this study.

Future work is clearly required to establish whether targeting SEMA3A signalling would be beneficial to treat neovascular eye disease, and whether exogenous delivery or blockade of endogenous SEMA3A is more useful. Moreover, it would need to be determined whether the SEMA3A pathway would be best targeted at the level of the ligand or its receptor(s) and examined whether anti-SEMA3A therapy would be well tolerated. Whether anti-NRP1^A^ antibodies that block SEMA3A-NRP1 interactions might also be utilised to treat neovascular eye pathology has yet to be assessed.

#### 6.2.2 Therapeutic potential of targeting ECM signalling through NRP1

NRP1 also promotes OIR-associated neovascularisation by mediating ECM-driven ABL1 activation (Raimondi et al., 2014). Should this pathway be found to contribute to human neovascular disease, a potential therapeutic intervention may already be available in the form of Imatinib. Thus, it is conceivable that Imatinib may be repurposed from its original use as an FDA-approved drug for cancer treatment (www.fda.gov) as a drug to treat neovascular eye disease. When administered systemically, as in cancer treatment, Imatinib can cause bone marrow suppression due to its potent inhibition of the hematopoietic stem cell regulator KIT (Growney et al., 2005). However, intravitreal delivery of Imatinib in ocular pathologies may circumvent this and other systemic side effects. A first step to assess the safety and efficacy of intravitreal Imatinib delivery for ocular neovascular disease might involve follow-up of vascular normalisation in rodent models of OIR and CNV combined with an investigation of neuroretinal health and function after treatment.

### 6.3 Co-targeting of SEMA3A- and VEGF-signalling through NRP1

The intravitreal administration of a NRP1-derived ligand trap, effective for both SEMA3A and VEGF164, reduced myeloid infiltration and pathological neovascularisation in the OIR model (Dejda et al., 2014). However, further work is required to determine whether normalisation of endothelial cells, myeloid cells or both explains the beneficial effect of sequestering NRP1 ligands in attenuating neovascularisation. Alternatively, soluble NRP1 might be used to curb SEMA- and VEGF-dependent signalling in neovascular diseases. Recent evidence showed that soluble NRP1 inhibits VEGF activity. Thus, genetically modified mice expressing a soluble NRP1 form in the skin have a normal number of blood vessels with only a mild reduction in the lumen size, but strongly reduced vascular leakage after intradermal VEGF injection (Mamluk et al., 2005). In agreement, soluble S_12_NRP1 has antitumor activity when overexpressed in prostate cancer cells injected into rats (Gagnon et al., 2000, Schuch et al., 2002). Moreover, quiescent murine hepatocytes express high levels of soluble NRP1, which is transiently downregulated after hepatectomy, and this correlates with endothelial cell signalling to facilitate angiogenesis for liver regeneration (Panigrahy et al., 2014). It has not yet been examined whether the exogenous delivery of soluble form of NRP1 might also ameliorate neovascular eye diseases.

### 6.4 Micro RNA (miRNA)-mediated modulation of NRP1 expression

The discovery of miRNAs that regulate NRP1 expression (Bai et al., 2015, Liu et al., 2015, Peng et al., 2014, Wu et al., 2014) is a relatively new finding that may catalyse the development of novel NRP1 therapeutics to target several different NRP1 functions in parallel. miRNAs are small non-coding RNAs that regulate gene expression by hybridising to sequences in mRNAs, most commonly within in the 3′ untranslated region, to degrade them or block their translation (Dangwal and Thum, 2014). miRNA mimics are synthetic RNA molecules that are designed according to template miRNAs to mimic their function (reviewed in Bader et al., 2010). The first miRNA mimic, MRX34, has been brought to clinical trial with the eventual aim of targeting liver cancer by mimicking miR-34a's regulation of a plethora of oncogenes (Bouchie, 2013). The miRNAs miR-338 and miR-320 are amongst the miRNAs that have been shown to downregulate NRP1 expression, and their endothelial expression is associated with reduced angiogenesis *in vitro* and in tumour xenograft models (Peng et al., 2014, Wu et al., 2014). Although it remains to be determined whether NRP1-targeting miRNAs could be utilised to treat eye disease, the broad specificity of miRNAs might be advantageous to downregulate gene expression programmes that drive pathology. However, this same property may also increase the risk of unexpected and unwanted side effects.

## 7 Future directions

VEGF blockade is now first line therapy for the treatment of macular oedema in ischemic retinopathies. In the case of exudative AMD, anti-VEGF therapy stabilises sight in over 90% of patients over several years of treatment (Rosenfeld et al., 2011), whereby the agent is administered by repeated monthly injection into the eye to maintain the beneficial effect (Schmidinger et al., 2011). Moreover, 30% of patients experience improved vision after anti-VEGF delivery (Rosenfeld et al., 2011). However, a significant number of patients do not benefit from anti-VEGF, and a multi-cohort clinical study examining the efficacy of long-term anti-VEGF treatment showed that only one third of patients had a good visual outcome at seven years, whilst another third had a poor outcome (Rofagha et al., 2013).

Inefficacy of anti-VEGF therapy may result from the treatment's inability to target established vascular lesions and/or prevent the emergence of new lesions driven by VEGF-independent angiogenesis pathways, such as ECM-induced angiogenesis. In addition, several rodent studies suggest that blockade of all VEGF isoforms in mouse models increases neuronal death in the mouse retina (Nishijima et al., 2007, Park et al., 2014). As most anti-VEGF drugs sequester all VEGF isoforms, neuroretinal degeneration due to a loss of trophic VEGF effects may, at least in part, contribute to the poor visual outcome observed with long-term treatment. Whilst comparable long-term data are not available for anti-VEGF treatment of DME, it is reasonable to suspect that similar pitfalls exist.

As long-term anti-VEGF monotherapy for exudative AMD has limited efficacy, does not work in all patients and has potential neuronal side effects, there is a need for alternative treatments for this and possibly other ischemic eye diseases. An alternative strategy to treat neovascular disease may be to preferentially target VEGF165 over other VEGF isoforms in circumstances where preserving VEGF120 signalling through VEGFR2 may be beneficial, especially for the long-term treatment of chronic diseases such as exudative AMD. Here, wherein blockade of all VEGF isoforms would likely increase neuronal death in the retina, preservation of VEGF120 may be sufficient to convey neuroprotection through signalling to VEGFR2-expressing neurons (Foxton et al., 2013, Nishijima et al., 2007, Saint-Geniez et al., 2008). In this respect, targeting VEGF164 binding to NRP1 might have similar effects as treatment with Macugen, an aptamer that specifically targets VEGF164 and has been approved by the FDA for the treatment of AMD, even though its clinical use has been superseded by anti-VEGFs that target all isoforms (Ip et al., 2008, Rosenfeld, 2006).

As NRP1 regulates angiogenesis and permeability induced by multiple signals, including VEGF, ECM and semaphorins, targeting NRP1 pathways may offer numerous windows for intervention in eye disease. Yet, it is the characteristics of the individual pathologies that should govern considerations on which type of NRP1-based targeting approach might be explored. For example, a total blockade of NRP1 signalling might be advisable when there is a need to block VEGF-dependent, VEGF-independent and SEMA3-dependent signalling through NRP1. Alternatively, it may be preferable to exclusively target VEGF-dependent and SEMA3-dependent NRP1 signalling, whilst leaving ECM/NRP1-driven angiogenesis untouched to harness NRP1's positive roles in facilitating vascular tissue integration and thereby restoring blood flow to hypoxic tissues. More specifically, it may be desirable to promote NRP1-mediated signalling to guide vessels along ECM remnants of degenerated vasculature whilst blocking harmful NRP1 functions, such as its ability to promote vascular hyperpermeability. With NRP1 emerging as a promising therapeutic target for eye disease and with encouraging therapeutic agents being developed, further studies are clearly needed to define the specific mechanisms by which NRP1 activates different disease-related pathways and to hone in on pathology-specific applications.
